
JOURNAL INFORMATION
==============================
NLM Title Abbreviation: Eur Psychiatry
Journal ID: Eur Psychiatry
NLM Journal ID: 9111820
Journal ID: EPA

European Psychiatry

ISSN: 0924-9338
EISSN: 1778-3585
Publisher: Cambridge University Press

ARTICLE INFORMATION
==============================
PMCID: PMC9412722
DOI: 10.1192/j.eurpsy.2020.10
Article ID: S0924933820000103
Article version: 1
Subjects: Abstracts

Symposium

Publication date: 2020 Jul
Electronic publication date: 2020 Sep 4
Volume: 63
Issue: Suppl 1
Issue title: Abstracts of the 28th European Congress of Psychiatry
First page: S597
Last page: S617
Copyright: © The Author(s) 2020
Copyright year: 2020
Copyright holder: The Author(s)
License: This is an Open Access article, distributed under the terms of the Creative Commons Attribution licence (http://creativecommons.org/licenses/by/4.0/), which permits unrestricted re-use, distribution, and reproduction in any medium, provided the original work is properly cited.
License URL: https://creativecommons.org/licenses/by/4.0/

Page count: 21

==============================

### Cognitive impairment across the schizophrenia spectrum: focus on assessment and treatment

### Abstracts

#### S005

#### The impact of cognitive and social cognitive impairment on real-life functioning in subjects with schizophrenia

G. Sachs[1]* and A. Erfurth[2]

Cognitive impairment is present since the first manifestations of the disorder, including social cognition in subjects at ultra-high risk for psychosis (UHR) [1]. While progress in psychopharmacology in the last decades has clearly changed the course of the disease, antipsychotic therapy is more efficacious in the treatment of positive symptoms than in the treatment of negative symptoms, of comorbid depression and of cognitive and social cognitive dysfunction [2]. Also because of this, cognitive impairment has a significant impact on functional outcome, greater than symptom severity. The relationship of facial recognition deficits and cognition in schizophrenia has been described early on, including its impact on functional outcome [3]. Since then, a large body of literature has shown that cognitive dysfunction in patients with schizophrenia accounts for 20-60% of the variance in measures of outcome and that in many studies cognition is more closely related to functional outcome than positive or negative symptoms [4]. A network analysis investigating 740 patients with schizophrenia [5] found that functional capacity and everyday life skills were the most central and highly interconnected nodes in the network. In summary, cognitive dysfunction plays a pivotal role as enduring problem after clinical remission and as critical rate-limiting factor in functional recovery. [1] Modinos G et al. JAMA Psychiatry. 2019. doi: 10.1001/jamapsychiatry.2019.3501. [2] Haddad PM, Correll CU. Ther Adv Psychopharmacol. 2018; 8(11):303-318. [3] Sachs G et al. Schizophr Res. 2004; 68(1):27-35. [4] Strassnig M et al. J Psychiatr Res. 2018; 104:124-129. [5] Galderisi S et al. JAMA Psychiatry. 2018; 75(4):396-404.

#### S008

#### New Developments of Cognitive Remediation in Schizophrenia

T. Wykes

We have known that people with a diagnosis of schizophrenia have cognitive problems and that they are related to future recovery. But despite this knowledge it has taken the academic and clinical communities a long time to recognise the potential for cognition to be improved through targeted treatment. The model was that the cognitive improvements generated could then benefit recovery and the attainment of personal goals. This treatment is called cognitive remediation. Its effectiveness has been demonstrated but we have not yet persuaded service providers to adopt it. One obvious question is "why?". There are still gaps in the evidence base which may improve effectiveness which will increase the benefits in terms that service providers and service users' value. Some of these new developments are based on factors thought to be important to learning such as metacognition. This may boost learning and help with the translation of within therapy benefits to everyday life. Some of these potential benefits will be presented. The other issue is to understand the treatment effects especially fulfilling individual recovery goals. These are not rocket science and only require extra and least burdensome (on participants) measures to be added to studies. We also need to use data we already possess to carry out further analyses to indicate how different therapies work and to compare across studies in large individual participant level

EUROPEAN PSYCHIATRIC ASSOCIATION

databases. This is not a pessimistic but a pragmatic one on the next steps for delivering therapy to those who will reap the most benefit.

### Transdiagnostic Perspective of ADHD Among Different Mental Disorders

#### S009

#### ADHD and Obesity: Dopaminergic Signaling as Biological Link

N. Roth Mota[1]*, G. Poelmans[1], M. Klein[1], B. Torrico[2], N. Fernàndez-Castillo[2], B. Cormand[2], A. Reif[3], B. Franke[1] and A. Arias Vásquez[1]

Attention-Deficit/Hyperactivity Disorder (ADHD) and obesity are frequently comorbid, genetically correlated, and share brain substrates. The biological mechanisms driving this association are unclear, but candidate systems, like dopaminergic neurotransmission and circadian rhythm, have been suggested. Our aim was to identify the biological mechanisms underpinning the genetic link between ADHD and obesity measures and investigate associations of overlapping genes with brain volumes. We tested the association of dopaminergic and circadian rhythm gene sets with ADHD, body mass index (BMI), and obesity (using GWAS data of N=53,293, N=681,275, and N=98,697, respectively). We then conducted genome-wide ADHD-BMI and ADHD-obesity gene-based meta-analyses, followed by pathway enrichment analyses. Finally, we tested the association of ADHD-BMI overlapping genes with brain volumes (primary GWAS data N=10,720–10,928; replication data N=9,428). The dopaminergic gene set was associated with both ADHD (P=5.81x10-3) and BMI (P=1.63x10-5), the circadian rhythm was associated with BMI (P=1.28x10-3). The genome-wide approach also implicated the dopaminergic system, as the Dopamine-DARPP32 Feedback in cAMP Signaling pathway was enriched in both ADHD-BMI and ADHD-obesity results. The ADHD-BMI overlapping genes were associated with putamen volume (P=7.7x10-3; replication data P=3.9x10-2) – a brain region with volumetric reductions in ADHD and BMI and linked to inhibitory control. Our findings suggest that dopaminergic neurotransmission, partially through DARPP-32-dependent signaling and involving the putamen, is a key player underlying the genetic overlap between ADHD and obesity measures. Uncovering shared etiological factors underlying the frequently observed ADHD-obesity comorbidity may have important implications in terms of prevention and/or efficient treatment of these conditions.

#### S012

#### Investigating the effects of physical activity on positive and negative affect in the everyday life of patients with adhd – a mobile health approach

E. Koch[1]*, C. Freitag[2], J. Mayer[2], A. Reif[3], O. Grimm[4], J.A. Ramos-Quiroga[5], P. Ibáñez[5], J. Palacio[5], J. Kuntsi[6], A. Pawley[6], J. Buitelaar[7], D. Bergsma[7] and U. Ebner-Priemer[1]

**Introduction:** Physical activity is beneficial for both physical and mental health. Thus, several Ambulatory Assessment studies have investigated the association between physical activity and affect, but mainly in healthy individuals. Individuals with mental disorders, e.g., ADHD, rarely have been subjects of Ambulatory Assessment studies investigating this association.
**Objectives and Methods:** To investigate affect-responses to physical activity in the everyday life of patients with ADHD and healthy controls, we used multilevel-models to analyze data from electronic diaries and accelerometers (N=118 individuals, aged 14-44 years). Participants reported on positive and negative affect repeatedly in real-time via Smartphone-App and wore accelerometers across four days.
**Results:** Preliminary findings show that physical activity increases positive affect and decreases negative affect in the short term in healthy individuals. Patients with ADHD show similar patterns, with minor differences to the healthy control group.
**Conclusions:** Physical activity influences both positive and negative affect in patients with ADHD and healthy individuals. Translated into practice, these findings may serve as an empirical basis for future interventions targeting the improvement of affective instability in patients with ADHD.

### Understanding the variation of the human brain: genes, sex, environment and psychopathology

#### S023

#### Does fetal testosterone differently influence male and female brain development?

A. Ruigrok[1]*, R. Holt[1], N. Dooley[1], M. Lombardo[2], B. Auyeung[3] and S. Baron-Cohen[1]

There are average differences in prenatal sex steroid hormone levels, as higher levels of fetal testosterone (FT) are needed for male sexual differentiation. Previous research has shown that FT was associated with gray matter (GM) brain regions that show on average volumetric sex differences in prepubertal development. On average, in 8-11 year old children, GM volume in right temporal parietal junction posterior superior temporal sulcus was greater for boys compared to girls and, as expected, positively predicted by FT. On the other hand, GM in the planum temporal/parietal operculum was greater in girls compared to boys and was negatively predicted by FT. Here we will discuss whether FT differently affects male and female brain development in a typically developing cohort. We will examine (1) if and how FT is associated with thickness and surface area in the male and female adolescent brain and (2) if there is overlap between FT associations and overall sex developmental differences in an independent cohort, matched on age and pubertal status.

### A new model to manage difficult to treat depression

#### S032

#### Is bipolar depression a different type of depression?

A. Fiorillo

Depressive episodes in patients affected by bipolar disorders are often misdiagnosed, especially in the case of a depressive onset of the disorder. This misdiagnosis has several clinical, therapeutic and prognostic implications, and there is the need for clinicians to be trained in correctly differentiating bipolar depression from other types of depression. In fact, bipolar depression has different genetic, clinical and therapeutic characteristics from unipolar depression. In particular, bipolar depression is thought to have a higher genetic loading, the age at onset is younger in bipolar depression and the affective episodes tend to be shorter and more frequent. From a therapeutic perspective, patients with bipolar depression require a specific treatment plan, which is different from that for unipolar depression. In particular, the use of antidepressant agents in these patients is debated given the high risk of switching between the two polarities and of suicidal behaviours. Strategies should be adopted to improve the clinicians' skills to identify, diagnose and treat bipolar depression.

### Etiopathogenesis of suicidal behaviour and major depression: novel approaches

#### S036

#### Genomics of suicidal behaviour

P. Courtet

Genetics constitute a substantial component of suicide risk. Epidemiological studies provide strong evidence for a genetic component to suicidal behavior, with a heritability estimate of 30%. The identification of biological factors contributing to the risk for suicidal behavior is an important topic of investigation in order to facilitate the identification of relevant targets for suicide prevention. Genetic, epigenetic, and expression studies have identified multiple promising genetic variants, candidate genes, and biological pathways. We will present here data from both, large-scale genome-wide studies and exploratory analyses from candidate genes studies, in different suicidal phenotypes. 1) We performed a GWAS of a population of 2 446 suicide attempters by using a Illumina “Global screen array“ for 687 572 SNPs. 2) A whole Methylome has also been realized in depressed patients with and without a history of suicide attempt stratified on the history of childhood abuse, using a Illumina Infinium® Human Methylation450. Results for potential genetic or methylomic markers of suicide attempt and / of early maltreatment will be discussed. 3) Pain being identified as highly related to suicidal behaviour, the involvement of opioidergic system in suicidal process opens new therapeutic strategies, particularly mu opioidergic agonists, in treating suicidal ideation. We will present results from association studies focusing mu and kapa opioid receptor genes, suggesting while exploratory, that both suicidal ideation and treatment emergent suicidal ideaion would be associated with the a mu opioid receptor functional polymorphism. By embracing complementary genomic approaches, it is hoped that the pathophysiological understanding of suicidal behaviour can be improved to justify the development of specific therapies.

### E-mental health across the lifespan

#### S041

#### Mindfulness and virtual reality in adults with adhd: results of a randomised, controlled clinical trial

J.A. Ramos-Quiroga

**Introduction:** Attention deficit hyperactivity disorder (ADHD) is a highly prevalent neurodevelopmental disorder, which presents a high comorbidity with anxiety and affective signs and symptoms. It has repercussions on the functioning of those suffering from it, who also have low therapy compliance and generate a significant cost both at a personal level and for society. Mindfulness is a psychological treatment that has proved to be effective for ADHD. Virtual reality is widely used as treatment in cases of phobias and other pathologies, with positive results.

**Aims:** To develop the first treatment for ADHD in adults based on virtual reality and mindfulness, while also resulting in increased treatment adherence and reduced costs.

**Patients and Methods:** We conducted a pilot study with 25 patients treated by means of virtual reality, in four 30-minute sessions, and 25 treated with psychostimulants. Measures will be taken pre-treatment, post-treatment, to evaluate both ADHD and also depression, anxiety, functionality and quality of life. Data will be later analysed with the SPSS v. 20 statistical program. An ANOVA of independent groups will be performed to see the differences between treatments and also a test-retest to detect whether the changes will be maintained.

**Results and Conclusions:** It is necessary to use treatments that are effective, reduce costs and increase therapy adherence. Treatment with virtual reality is an interesting alternative to the classical treatments, and is shorter and more attractive for patients.

**Disclosure:** J.A.R.Q was on the speakers' bureau and/or acted as consultant for Eli-Lilly, Janssen-Cilag, Novartis, Shire, Lundbeck, Almirall, Braingaze, Sincrolab, Medice and Rubió in the last 5 years. He also received travel awards (air tickets + hotel) for taking p

### S042

### The super brains app for adhd

S. Kooij

ADHD is a.o. characterised by forgetfulness and planning difficulties, leading to a chaotic lifestyle. In order to empower patients, organisational skills should be digitally supported before, during and after treatment. Rutger den Hollander, an expirience expert and owner of an ICT company, and Sandra Kooij, psychiatrist, developed the Super Brains app for this purpose. Patients can use the app on their own to learn more about ADHD, to contact communities, as well as for selfmanagement and lifestyle interventions. The app also intends to increase treatment effectiveness in blended care. Super Brains contains several functions such als self-tests, psycho-education, CBT video's, tools for the development of habits and routines, and (video) chatt contact with communities, as well as the therapist. All activities that contribute to personalised goals, as well as helping others, are rewarded using gamification. Super Brains was launched January 2020 at PsyQ, psycho-medical programs in the Netherlands. Already 6000 people have shown interest. We will study the feasability, effectiveness and consumer satisfaction of the app. We will also study using questionnaires and wearables, the mental and physical condition of users over time. During this presentation, first experiences using the app in blended care for adults with ADHD will be presented.

**Conflict of interest:** No

**Keywords:** Adult; ADHD; eHealth; Super Brains

### S045

### Kynurenine pathway, inflammation and schizophrenia

A. Szulc

There is a great variety of immune alterations observed in the peripheral blood or cerebrospinal fluid of patients with schizophrenia that include elevated levels of pro-inflammatory cytokines, specific and non-specific antibodies, components of complement cascade, acute phase proteins as well as shifts in the subpopulations of lymphocytes. There is also a growing literature suggesting that up-regulation of immune response is related to alterations of kynurenine pathway function and together are involved in pathogenesis of schizophrenia. Kynurenic acid (KynA) is a neuroactive metabolite of tryptophan formed in the brain and in the periphery, known to block ionotropic glutamate receptors and α7 nicotinic receptors, and to act as a ligand of G protein-coupled GPR35 receptors and human aryl hydrocarbon (AHR) receptors. Since KynA is the only known endogenous NMDA receptor antagonist, psychosis and schizophrenia were postulated to be caused by the effect of elevated KynA on glutamatergic and ultimately, dopaminergic neurotransmission Central level of KynA may increase in the course of inflammation, which is consistent with the inflammatory hypothesis of schizophrenia. The reported increase in KynA in schizophrenia and psychosis may originate from inflammation-driven increases in KYN (kynurenine). As in the case of mood disorders, there is evidence that inflammation may play a mechanistic role in schizophrenia and PET studies have reported increased microglial activation in patients with schizophrenia Alterations of immune response and disturbed functioning of kynurenine pathway may lead to disproportion between neuroprotective and neurotoxic mechanisms in the brain.

**Conflict of interest:** No

**Keywords:** kynurenine pathway; schizophrenia

### S048

### Unravelling immune alterations associated with the deficit schizophrenia subtype

J. Samochowiec[1*], B. Misiak[1,2], J. Kucharska-Mazur[1] and M. Wroński[1]

*Corresponding author.

Goal of the ongoing work is to understand the dynamic interactions of neuroimmune components in the transition from normal brain function to dysfunction at the molecular, cellular levels in patients with schizophrenia especially deficite type. We have recruited and examined n=195 Non-deficit and n= 309 Deficit schizophrenia patients. In this study, we tested whether schizophrenia patients at different stages of illness might present alterations in the levels of

HERV-K methylation. No significant differences in HERV-K methylation levels between D-SCZ and ND-SCZ as well as HCs were found. Our results indicate lower HERV-K methylation levels at early stages of schizophrenia. This difference might normalize with subsequent exacerbations of schizophrenia, likely due to the effects of anti- psychotics. Additionally we have tested a hypothesis whether variation in immune-inflammatory genes (IL-6, TGF-β, CTLA-4 and CD28) might be associated with susceptibility to deficit schizophrenia.Polymorphisms in immune-inflammatory response genes are believed to impact schizophrenia susceptibility.However, it remains unknown whether immunogenetic factors play a role in the etiology of deficit schizophrenia (D-SCZ). Therefore, we genotyped four polymorphisms in genes encoding two immune system regulatory proteins (CTLA-4 rs231775 and CD28 rs3116496), interleukin-6 (IL6 rs1800795) and transforming growth factor-β (TGFB1 rs1800470) in 513 schizophrenia patients and 374 controls. The CD28 rs3116496-CC genotype and C-allele were significantly more frequent in the whole group of patients and D-SCZ patients compared to controls. Our results indicate that the CD28 rs3116496 polymorphism might impact the risk of schizophrenia, especially D-SCZ. Immune-inflammatory alterations hold a great promise for better understanding disease etiopathology

### Psychiatric comorbidities in bipolar disorders: double penalty and trajectories

#### S051

#### The role of substance abuse and polygenic risk in the early course of bipolar disorder

T.V. Lagerberg

Comorbid abuse of cannabis or alcohol is highly prevalent in bipolar disorder, as is daily use of tobacco. However, the role of use of these substances in the early course of the disorder is not well explored. For some individuals, the abuse develops premorbidly, while for others it may develop as a secondary complication with potential for prevention. It is, however, not well known how substance abuse influences the early clinical course of bipolar disorder. In the current study, we explore the relationship between substance abuse and clinical course in a first treatment one-year follow-up study of patients with bipolar I, II and NOS disorder, recruited to the TOP Study in Oslo, Norway. We investigate substance abuse diagnoses as well as continuous measures of substance use. Since polygenic risk for bipolar disorder appears to be elevated in individuals with comorbid cannabis use and may influence clinical features, this will be considered when investigating how substance abuse including nicotine use affects clinical evolution including early relapse and suicidal behaviour.

#### S052

#### Psychopathological outcomes of clinically relevant adolescent borderline personality disorder symptoms

S. Marwaha

**Objectives:** This study examined associations between adolescent borderline personality disorder symptoms and subsequent depressive, psychotic and hypomanic symptoms.
**Methods:** We used data from the Avon Longitudinal Study of Parents and Children. Participants were adolescents living in the community who had data for all longitudinal outcomes (N = 1758). We used logistic regression and path analysis to investigate associations between borderline personality disorder reported at age 11–12 years and depressive and psychotic symptoms reported at age 12 and 18, and lifetime hypomanic symptoms reported at age 22–23 years.
**Results:** Adolescent borderline personality disorder symptoms were associated with psychotic symptoms (odds ratio: 2.36, confidence interval: [1.82, 3.06]), diagnosis of depression at age 18 years (odds ratio: 1.30, confidence interval: [1.03, 1.64]) and hypomanic symptoms (odds ratio: 2.89, confidence interval: [2.40, 3.48]) at 22–23 years. Path analysis controlling for associations between all outcomes indicated that borderline personality disorder symptoms were independently associated with depressive symptoms ($\beta = 0.97$, $p < 0.001$) at 12 years and hypomanic ($\beta = 0.58$, $p < 0.01$) symptoms at 22–23 years. Borderline personality disorder symptoms were also associated with psychotic symptoms at age 12 years ($\beta = 0.58$, $p < 0.01$), which were linked ($\beta = 0.34$, $p < 0.01$) to psychotic symptoms at age 18 years.
**Conclusion:** Adolescents with borderline personality disorder symptoms are at future risk of psychotic and hypomanic symptoms, and a diagnosis of depression. Future risk is independent of associations between psychopathological outcomes, indicating that adolescent borderline personality disorder symptoms have multifinal outcomes. Increasing awareness of borderline personality disorder in early adolescence could facilitate timely secondary Prevention of these symptoms subsequently, helping to prevent future psychopathology.

### Psychosocial imaging: disentangling the interplay between environmental variables and psychotic disorders

#### S053

#### The causal role of environmental factors in psychotic disorders with a focus on urban living

J. Kirkbride

Urban living has been associated with increased risk of psychotic disorders, particularly schizophrenia, in several studies in the 20th century and early 21st century, particularly in high income countries in the Global North. New data and methods, emerging from both the Global South and North, provide new directions for understanding whether being born, brought up in or living in urban areas is causally related to future risk of psychotic disorders. In this talk I highlight and review these new and emergent trends, drawing on research published in Sweden, the UK, Chile and low and middle income countries. This literature suggests more complex patterns of association between urban living and psychosis, which nonetheless may have critical clues for both aetiology and public mental health responses to global variation in the incidence of psychotic disorders.

### S054

### The complexity of vulnerability to psychosis

A. Fiorillo

Schizophrenia is a complex mental disorder, which has been recently conceptualized as a neurodevelopmental disease. This conceptualization has changed the psychopatological approach to schizophrenia, which is now described as lying on a continuum from mild psychotic experiences to frank psychotic episodes. According to this theory, the presence of psychotic symptoms would represent the final pathway of a complex dysregulation and interaction of different genetic and environmental risk factors. As regards genetic liability, recent genome-wide association studies have identified a total of 108 loci containing common risk alleles, and which meet genome-wide significance. As regards environmental factors, higher rates of schizophrenia have been found in ethnic minority groups, in persons who are heavy cannabis smokers, in those who suffered from severe childhood traumas, in persons who have been reared in highly deprived settings. The identification of risk factors associated with vulnerability to psychosis is essential for improving our understanding and early detection of vulnerable individuals, and to propose tailored and timely interventions for sufferers. There is the need for an interdisciplinary approach to schizophrenia which includes screening procedures for individuals reporting specific vulnerabilities and treatment strategies tailored on patients' needs.

## Bipolar disorders on top of mood stabilisers: can we improve the general health?

### S057

### Focus on metabolic features associated with bipolar disorders

S. Jaworowski[1] and I. Pacchiarotti[2]*

There is evidence suggesting that individuals suffering from BD are at increased risk for somatic diseases in comparison to healthy controls. Among the clinical diseases that are comorbid with BD, there are high rates of obesity, hypertension, dyslipidemia, type 2 diabetes mellitus and metabolic syndrome.According to literature reviews, diabetes mellitus occurs up to three times as often among individuals with BD, as it does in the general population. Obesity is also prevalent, and glucose and lipids are dysregulated at high rates in patients with BD. The exact relationship between metabolic disturbances and BD is very complex and so far still not well understood. BD has shown to increase the odds of rehospitalization for non-mental conditions, which often remain underdiagnosed and undertreated in this context (Dickerson et al., 2016). Some studies have associated the presence of type 1 or 2 diabetes mellitus with a higher risk of mortality among the physical comorbidities in patients with BD.Preliminary investigations suggests that obesity and Metabolic issues might negatively impact the course of illness in BD. Preliminary investigations suggests that obesity and MetS might negatively impact the course of illness in BD. Since research pointing at the potential negative implications of metabolic issues in clinical outcomes in BD is growing, this symposium aims at assessing the existing literature on the impacts of metabolic disturbances on the frequency of affective episodes, hospitalizations, cognitive impairment, suicidal behavior, global functioning and other potential outcomes in BD.

**Disclosure:** Dr Pacchiarotti has received CME-related honoraria, or consulting fees from ADAMED, Janssen-Cilag and Lundbeck.

### S058

### Innocence faded: childhood adversity and the hippocampus in healthy and bipolar individuals

D. Janiri

Sapienza University, Psychiatry, Rome, Italy

Life events can significantly influence the clinical presentation of mood disorders. In particular, childhood trauma is a relevant environmental stressor associated with risk for both type I and type II bipolar disorders (BD). Childhood trauma can influence BD clinical outcome by inducing earlier disease onset, a greater number of episodes, a rapid cycling course and by increasing the number of hospitalizations. Furthermore, several studies consistently showed that early adverse events are strongly associated with increased risk for suicidal acts. The hippocampus is involved in the biological response to stress, as it is implicated in the processing of traumatic memories and in emotional response to life events. In non-clinical population childhood trauma has been associated with lower hippocampus volumes. Considering hippocampal anatomical complexity, previous studies showed this effect in specific hippocampal subfields. These results have not been replicated in patients with BD, suggesting a differential impact of early adverse events on hippocampal subfield volumes of patients with BD and healthy individuals. A specific interaction between childhood trauma and BD diagnosis could modulate differences on those hippocampal subfields involved in emotion regulation. This presentation will focus on the relationship between childhood trauma and BD and on its great clinical relevance. The latest data on biological effects of childhood trauma on hippocampal volumes will be discussed.

### S059

### Sleep in bipolar disorders: a prodrome, a symptom, a treatment?

A. Murru

Bipolar disorder (BD) presents differences in sleep architecture and circadian rhythms compared with the general population. Sleep disturbances represent a constant element in the syndromic definition of mood episodes, and are contemplated in both the DSM and the ICD classifications. Manic states are generally associated with a decrease in the need for sleep or insomnia, while bipolar depression is associated with symptoms of insomnia or hypersomnia. Sleep-Wake, Energy and Movement Alterations are also highly prevalent in euthymic phases of BD, present in around 30%-80% of BD patients, depending on the definitions used and the management of potential confounding factors, such as drug regimen. Sleep alterations are a frequent occurrence also in interepisodic phases of BD, and they have been also described both long before BD onset, as unspecific risk syndromes, or as immediate prodromes of BD onset. In fact, sleep disturbances may frequently appear months or years before the onset of the affective syndrome, being often present during childhood/adolescence. A decreased need for sleep may anticipate the onset of a manic episode, whilst insomnia may anticipate acute episodes of both manic and depressive polarity. Hypersomnia seems to precede bipolar depressive episodes, but it has been linked also to mixed depressive episodes. Also, sleep alterations are specifically associated with an increased risk of suicidal behavior. This presentation will focus on the state of the art evidence on sleep alterations in bipolar disorders, focusing on the opportunities provided by new technologies in measuring sleep and activity alterations.

### S060

### Psychosocial interventions for patients with bipolar disorder and their relatives

A. Fiorillo[1] and M. Luciano[2*]

According to international guidelines for the optimal management of patients with bipolar disorder, psychosocial interventions should be provided to patients and their family members. Several psychosocial interventions are currently available, including cognitive behavioural therapy (CBT), cognitive remediation, psychoeducation, interpersonal and social rhythm therapy. As regards psychoeducation, the efficacy is stronger when it is provided in group format compared to the individual approach. The involvement of family members in the psychoeducational intervention leads to better results. Moreover, when psychoeducation is provided together with CBT the risk of manic symptoms is reduced, patient's personal functioning is increased and medication non-adherence is improved. Although psychosocial interventions have been proven to be effective in patients with bipolar disorders, their availability in routine clinical care is not satisfying. Several organizational difficulties have been identified, such as the lack of time and the lack of adequate training for mental health professionals. Other difficulties in the implementation of these approaches in bipolar disorder are related to the characteristics of the illness itself; patients are highly motivated to participate in the intervention during the depressive phases, but are reluctant to do it when they are in mania. In order to increase patients' adherence to treatment protocols, it may be useful to adopt new strategies (such as advance directives or reminders through smartphones) and to develop shorter psychosocial approaches.

## Negative symptoms in subjects with schizophrenia and at high risk for psychosis: the state of the art of assessment and treatment

### S061

### Main advantages and limitations of validated assessment instruments for negative symptoms

S. Dollfus[1,2]

Standardized assessments for negative symptoms are necessary in clinical practice, Research and therapeutic trials. As numerous scales have been developed, their advantages, limitations and some recommendations need to be described. Most of the scales are based on observer ratings but self-reports on negative symptoms have been recently developed. The NIMH-Negative Symptom Consensus Development Conference (Kirkpatrick,et al., 2006) has been a milestone for the development of a second generation of scales. These scales such as BNSS (Kirkpatrick et al., 2011) and CAINS (Blanchard et al., 2011) assess the 5 negative dimensions (alogia, social withdrawal, anhedonia, blunted affect and avolition) as recommended by the conference and also assess internal experiences for avolition and social withdrawal. Due to their good psychometric properties, they should be recommended for the assessment of negative symptoms. They supplant the first generation scales such as PANSS negative, SANS, NSA-16, SDS. These scales, although widely used, should not be recommended due to their limits according to the present concept on negative symptoms. However, SDS should be still considered for categorizing patients into deficit and no deficit sub-groups. Two recent self-assessment scales, the MAP-SR (Llerena et al., 2013) and the SNS (Dollfus et al., 2016) have been also developed. Due to the limits of first-generation self-assessments, they should be used for self-assessing negative symptoms as complementary measure of observer-ratings. Finally, other scales assessing anhedonia and avolition, initially developed in disorders other than schizophrenia, can also be considered since good psychometric properties have been observed in patients with schizophrenia.

**Disclosure:** S. Dollfus received honoraria as expert/consultant by Fabre, Gedeon; invited Conferences: Lundbeck, Otsuka, Janssen, and has contracts with Prophase MedAvances and NeuroCog-Trials.

### S064

### Evidence-based treatment of negative symptoms?

S. Kaiser

The negative symptoms of schizophrenia remain a major therapeutic challenge. Nevertheless, there is a growing evidence base regarding the effects of biological and psychosocial interventions on negative symptoms. This presentation will provide an overview on how this evidence base has been integrated in the formulation of the EPA guidance on the treatment of negative symptoms. Although the importance of the distinction between primary and secondary negative symptoms for treatment selection might seem evident, the current evidence based remains limited. Nevertheless, recommendations based on good clinical practice can be formulated. In the main part of the talk, interventions with a sufficient evidence to provide a recommendation are discussed, including antidepressant augmentation, social skills training and other psychosocial interventions. It is important to note that these recommendations for certain interventions are not specific for primary or predominant negative symptoms. In addition to the recommendations, the limitations of the current evidence base and directions for future research will be discussed.

**Disclosure:** SK has received royalties for cognitive test and training software from Schuhfried, Austria.

## Intercultural opening of mental health care services across europe

### S067

### Do asylum seekers and refugees need specialised services? pitfalls and challenges of mental health care for asylum seekers and refugees. status quo in europe

L. Küey

This article discusses a current dilemma in the mental health care for refugees and asylum seekers: specialized or mainstream services? The last decade had witnessed an increasing number of forcibly displaced people settling in European countries. Many are in the status of refugees, fewer in asylum seeking status. The integration process also includes mental health issues. Accordingly, Health and mental health care systems are facing a dilemma: whether constructing new specialized services for refugees and asylum seekers or integrating such services into the mainstream services. Creating special services has the advantage of responding to the unique needs of this population groups. The high prevalence of complex traumas and related complex mental disorders shape a series of special mental health needs. On the other hand, such special services have a risk of marginalizing refugees and asylum seekers. However, organizing mental health care for refugees and asylum seekers in the mainstream health services faces a different challenge: how to respond the unique needs of this population groups becomes a crucial question. Training of the already available health / mental health personnel, or the health personnel who themselves are refugees are some of the solutions practiced so far. This is not only a process of facing new problems but also opens the doors for a more multi-cultural milieu both for the receiving society and the refugees, also in the fields of Health and mental health. It is also an opportunity of co-creating new ways and models of improving more culturally competent health systems.

**Conflict of interest:** No

## My dealer is a doctor: on latrogenic addictions

### S072

### Should we stimulate prescribing stimulants?

J.A. Ramos-Quiroga

Across all available guidelines and the European consensus statement, a multimodal treatment approach is recommended that addresses adult ADHD and associated co-morbid disorders in the treatment plan if symptoms are clinically impairing and involve the patients' partners and, family members. The multimodal treatment approach should include psycho-education, pharmacotherapy, and disorder-oriented psychotherapy for ADHD and family or couple therapy if needed. Pharmacotherapy for adults with ADHD has shown to be highly effective and is recommended as first-line treatment for adult ADHD in the NICE guidelines. The first-line drugs for adults with ADHD are the stimulants (methylphenidate or lisdexamphetamine). The effect size for stimulants in adults is also quite high (around 1.0). Several stimulants are available with methylphenidate (MPH) being the most extensively studied. MPH is available in different formulations including immediate-release (IR) tablets, extended-release (ER) tablets and the OROS MPH formulation. Other stimulant treatment options for adult ADHD include mixed amphetamine salts ER, dexmethylphenidate IR and ER, and lisdexamfetamine dimesylate. Stimulants have proven to be effective in adults with ADHD: around 70% of patients respond to the first stimulant prescribed to them, while around 90% respond to either the first or second stimulant prescribed. The treatment with stimulants are quite well tolerated in adults, but it is necessary to carry out a clinical examination prebious to prescription (blood tension, family history of sudden dead). Treatment with stimulants in adults with ADHD is associated with reductions in criminality, car accidents, suicide, depression and substance use disorder.

**Disclosure:** J.A.R.Q was on the speakers' bureau and/or acted as consultant for Eli-Lilly, Janssen-Cilag, Novartis, Shire, Lundbeck, Almirall, Braingaze, Sincrolab, Medice and Rubió in the last 5 years. He also received travel awards (air tickets + hotel) for taking p.

### Unconventional treatments with unique mechanistic targets for major depressive disorder

#### S073

#### Unconventional treatments with unique mechanistic targets for major depressive disorder: ketamine

G. Vazquez

The discovery of rapid mood-elevating effects of low doses of the phencyclidine-like NMDA antagonist ketamine is an historically intriguing development, particularly since there has been very little that is fundamentally new for the biological treatment of severe depression since the development of ECT in the 1930s, monoamine oxidase (MAO) inhibitors in the mid-1950s, monoamine transport-inhibitors since the late 1950s, and recent applications of some second generation antipsychotic agents to the treatment of unipolar and bipolar depression. The first controlled clinical trial of ketamine as an antidepressant was reported in 2000, based rationally on pharmacological theory and animal modeling. Since then, a considerable amount of clinical research has been reported on the use of ketamine for treatment of major depression, including several case reports and case series, and some controlled and uncontrolled trials. Despite growing evidence for antidepressant effects of ketamine in both unipolar and bipolar depression, mainly for cases of resistant to treatment depression (TRD) and temporary reduction of suicidal thinking, many questions and challenges remain. Many aspects of its clinical pharmacology, efficacy, dosing and optimal routes of administration, safety and abuse potential in mood-disorder patients remain uncertain. Therefore, we will present here the benefits and risks of repeated low dose ketamine infusions to reduce suicidal ideation and produce sustained antidepressant effects over 4 weeks of an open-label trial for TRD.

**Conflict of interest:** No

#### S075

#### Unconventional treatments with unique mechanistic targets for major depressive disorder: cannabinoids

A. Bahji

Globally, major depressive disorder affects nearly 300 million individuals and is a major source of disability. Despite the availability of effective antidepressant treatments, between 10 and 30% of patients will not experience symptomatic relief despite several adequate trials of medication and psychotherapy. Treatment-resistant depression, therefore, represents a key dilemma for health care providers and policymakers. The search for novel pharmacologic targets has stimulated interest in not only the glutamatergic system and ketamine but also the endogenous cannabinoid system. Increasingly, as countries allow cannabinoids to be used for medicinal purposes, clinicians will increasingly encounter cannabis use in practice—either recreationally or for potential therapeutic indications. In terms of biological plausibility, a potential role exists in the endocannabinoid system in reducing depressive symptoms. However, this must be tempered against the potential for adverse events and the development of dependence or tolerance to cannabinoid use. This presentation will review the current evidence for and against cannabinoid use in the treatment of depression.

**Conflict of interest:** No

### Major depression as an episodic illness: benefits of re-framing the disease course

#### S082

#### Heterogeneity of major depressive disorder versus postpartum depression

S. Kasper

Major depressive disorder (MDD) affects over 300 million people worldwide and is a leading contributor to suicide. MDD is a heterogeneous, relapsing/remitting disorder characterized by individual major depressive episodes (MDEs). Postpartum depression (PPD) is a temporally-defined MDE in women during pregnancy or up to one year after childbirth, which may appear more homogeneous due to the temporal definition, hormonal triggers, and restriction to reproductive age. MDEs are characterized by ≥2 weeks of symptoms and changes from previous levels of function. Formal diagnosis requires ≥5 of the following symptoms: depressed mood, anhedonia, loss of energy, feelings of worthlessness or guilt, impaired concentration, suicide ideation/attempt, and disturbances in appetite, sleep, or psychomotor activity, with at least one symptom being depressed mood or anhedonia. PPD may also be associated with difficulty breastfeeding and/or bonding with the infant, social isolation, and anxiety. Risk factors for MDD include genetics/family history of depression, personal medical history (mental or other), and environmental factors (stress, diet, socioeconomics, trauma). Additional risk factors in PPD include pregnancy associated factors, such as hormonal fluctuations and perinatal stressors. MDE pharmacotherapy relies heavily on monoaminergic agents, including selective serotonin re-uptake inhibitors and serotonin-norepinephrine re-uptake inhibitors, which can be associated with delayed response and modest effect size. More recently, in the United States only, brexanolone was approved for the treatment of adult women with PPD. Appropriate treatment of MDEs must consider patient risk factors and the spectrum of symptoms at presentation, in addition to available pharmacotherapies.

**Disclosure:** SK received grants, fees, and/or honoraria from Angelini, AOP Orphan Pharmaceuticals AG, Celgene, Eli Lilly, Janssen-Cilage Pharma GmbH, KRKA-Pharma, Lundbeck A/S, Mundipharma, Neuraxpharm, Pfizer, Sage Therapeutics, Inc., Sanofi, Schwabe, Servier, Shire.

### S083

### Why do we consider major depressive disorder to be a chronic illness?

E. Vieta

Major depressive disorder (MDD) is one of the largest contributors to disability worldwide, and has a global lifetime prevalence of over 10%. Although professional society guidelines describe MDD in terms of discrete major depressive episodes (MDEs), the evolution of antidepressant treatment has framed depression as a chronic, lifelong disease. In the early twentieth century, depression was considered an acute illness, but by the mid-twentieth century, longitudinal studies of tricyclic and other monoaminergic antidepressants indicated that depression can have a life-long, episodic course, with relapse and recurrence. In the early 2000s, the Sequenced Treatment Alternatives to Relieve Depression (STAR*D) study demonstrated that, using current standards of care, 75% of patients with MDD experience episodic depression, with periods of remission and recurrence. Furthermore, failure to achieve remission may lead to frequent relapse and ineffective re-treatment. Conversely, effective treatment to remission at the first episode may reduce progression to chronicity/treatment resistance. The onset of response and dosing recommendations associated with selective serotonin re-uptake inhibitors and serotonin-norepinephrine re-uptake inhibitors have furthered the chronic approach to depression treatment. Recently, a new focus on the development of potentially rapid-acting antidepressants has emerged, which could potentially alter the chronic treatment paradigm and provide an alternative, treat-as-needed approach for individual MDEs. In this hypothetical treatment scheme, acute treatment modalities could allow off-treatment periods, while potentially maintaining remission from symptoms. Implementation of an acute treatment paradigm would require early detection/identification of an MDE, which could potentially be facilitated by the adoption of digital monitoring tools in clinical practice.

**Disclosure:** Dr. Vieta has received grants and served as consultant, advisor or CME speaker for the following entities: AB-Biotics, Abbott, Allergan, Angelini, Dainippon Sumitomo Pharma, Galenica, Janssen, Lundbeck, Novartis, Otsuka, Sage, Sanofi-Aventis, and Takeda.

### S084

### Sage-217 clinical data in patients with a depressive episode in major depressive disorder

R. Lasser

**Background:** Over 4.5% of the global population is estimated to suffer from major depressive disorder (MDD) annually. A double-blind, randomized, placebo-controlled Phase 3 study (NCT03672175; MOUNTAIN) evaluated the efficacy and safety of 20mg or 30mg of SAGE-217, an investigational, oral, neuroactive steroid $GABA_A$ receptor positive allosteric modulator, compared with placebo in MDD.
**Methods:** Patients (N=581),18-65 years old, diagnosed with MDD of qualifying severity [Hamilton Rating Scale for Depression (HAM-D) total score ≥22, and Montgomery-Åsberg Depression Rating Scale (MADRS) score ≥32], were randomized 1:1:1 to SAGE-217 20mg, SAGE-217 30mg, or placebo for two weeks of dosing, with follow-up through 182 days. HAM-D was assessed throughout the study [change from baseline (CFB), response (reduction ≥50%), and remission (score ≤7)]. CFB at Day 15 was the primary endpoint. Safety and tolerability were assessed by the adverse event (AE) frequency and severity.
**Results:** SAGE-217 30mg did not achieve a statistically significant HAM-D CFB versus placebo at Day 15 (primary endpoint; 30mg, -12.6; placebo −11.2; p=0.115). At Days 3, 8, and 12, SAGE-217 30mg demonstrated statistically significant HAM-D CFB versus placebo (p≤0.018 for each time point). Post hoc analyses of SAGE-217 30mg patients with measurable drug concentration showed significant HAM-D CFB versus placebo at all time points through and including Day 15 (p≤0.048 for each time point). The most common AEs (≥5%) in either SAGE-217 group were headache, dizziness, somnolence, fatigue, diarrhea, sedation, and nausea.
**Conclusions:** SAGE-217 achieved rapid (by Day 3) improvements in depressive symptoms and demonstrated numerical reduction in HAM-D at Day 15.

**Disclosure:** RL is an employee of Sage Therapeutics, Inc., with stock/stock options.

### S085

### Brexanolone and sage-217 clinical data in patients with postpartum depression

K. Deligiannidis

**Background:** Postpartum depression (PPD) is a common complication during and after pregnancy. Efficacy and safety of brexanolone IV (BRX) and investigational SAGE-217, two neuroactive steroid $GABA_A$ receptor positive allosteric modulators, were evaluated in randomized, placebo-controlled trials (RCTs) in PPD (3 BRX, 1 SAGE-217).

**Methods:** Trial inclusion criteria were similar: adult women, ≤6 months postpartum, with PPD of qualifying severity. BRX trial randomization was 1:1 to BRX 90 μg/kg/h iv (BRX90):placebo in Studies A and C and 1:1:1 to 90:60 μg/kg/h iv:placebo in Study B over 60 hours with post-dosing follow-up through Day 30. For SAGE-217, patients were randomized 1:1 to 30mg capsules versus matched placebo for 14 days of oral, evening, outpatient dosing, with follow-up through Day 45. The primary endpoint was change from baseline in Hamilton Rating Scale for Depression (HAM-D) at dosing cessation (BRX, Hour 60; SAGE-217, Day 15) versus placebo. BRX90 efficacy was assessed using an integrated dataset. Adverse events (AEs) were reported.
**Results:** At the primary endpoints, BRX90 and SAGE-217 demonstrated HAM-D score improvements versus placebo (BRX, $p<0.0001$; SAGE-217, $p=0.0028$). Statistical separation persisted through Day 30 (BRX90, $p=0.0213$) or Day 45 (SAGE-217, $p=0.0027$). AEs occurring in ≥5% of BRX patients, ≥2x the rate of placebo, were sedation/somnolence, dry mouth, loss of consciousness, and flushing/hot flush. AEs occurring in ≥5% of SAGE-217 patients were somnolence, headache, dizziness, upper respiratory tract infection, diarrhea, and sedation.
**Conclusions:** BRX and SAGE-217 demonstrated statistically significant improvements in depressive symptoms versus placebo in pivotal PPD RCT and were generally well tolerated.
**Disclosure:** KD serves as a consultant to Sage Therapeutics, Inc., receives NIMH support and royalties from an NIH employee invention, and reports grants from Sage Therapeutics, Inc., awarded to the Zucker Hillside Hospital during the conduct of the brexanolone inject

### Suicide behaviour in different mental disorders: epidemiological findings and perspectives on suicide prevention

#### S087

#### Prevalence and incidence of suicidal ideation and behaviour in delusional disorder, schizophrenia and other related disorders

A. González-Rodríguez

Suicide is a public health problem and one of the leading causes of premature death in Western countries. Most studies suggest that mental disorders are associated with increased suicide risk, being affective and psychotic disorders the most common diagnostic groups. Available literature suggests that suicide risk in psychosis is higher at the earlier stages of the illness, and it decreases as people age. Several risk factors for suicide have been widely identified in patients with psychosis. Duration of illness, gender, duration of untreated psychosis, comorbid depression, hopelessness and previous history of suicide attempts have an impact on suicide risk among these populations. Particularly, recent works have reported that insight, comorbid depressive symptoms and age at onset of illness may be potential predictors of suicidal behaviour in delusional disorder. However, surprisingly, few epidemiological and clinical studies have investigated the suicide phenomenon in patients with delusional disorder. Thus, the Parc Taulí- Delusional Disorder Workgroup (Sabadell, Spain) has developed a line of research on the study of suicidality in delusional disorder. A recent systematic review carried by the team explored the frequency of suicidal ideation, suicide attempts and completed suicide in four groups of psychotic disorders (e.g. schizophrenia, schizoaffective disorder, first-episode of psychosis and delusional disorder). Results from this work will be also discussed in this presentation. In brief, patients with schizophrenia showed the highest rates of suicidal behaviour and completed suicide compared to the other groups. Future research is needed to predict suicide. Reducing suicide risk in this understudied population is mandatory.
**Disclosure:** Alexandre González-Rodríguez has received honoraria or/and travel costs for congresses from Janssen and Lundbeck-Otsuka.

#### S088

#### Pain and interoception: perspectives on suicide prevention

P. Courtet

Intolerable pain is often reported in suicide notes. Otherwise, the frequency of life events preceding a suicidal act is high, especially interpersonal difficulties. Such adversity is the source of psychological or social pain. At a neuroanatomical level, suicidal vulnerability is associated with dysfunctional insula activation during social exclusion, a region involved in social and physical pain processing. Social pain elicited by social exclusion or devaluation shares common neurobiological patterns with physical pain. Despite the complexity of its definition, higher psychological pain levels are associated with suicidal ideation and acts, and we reported a different modulatory effect of decision-making. The neural circuits involved in the suicidal behavior are also targeted by the inflammatory system and interoceptive pathways. We will discuss the perspectives offered by examining the inflammatory response to social stress in suicidal patients, as well as their interoceptive deficits. Analgesics are usually used to get relief from pain but are also frequently involved in suicidal overdoses. It has been shown that opioid analgesics are associated with an increased risk of suicide. Then, opioids may be used by suicidal patients to get relief from psychological/social pain rather than from physical pain. Involvement of opioidergic system in suicidal process opens new therapeutic strategies, particularly mu opioidergic agonists, in treating suicidal ideation. The approach of the suicidal issue by the angle of pain, social disconnection and the embodied pain, offers new advances to improve clinical assessment, to identify new biological pathways involved in suicidal risk, and to propose innovative therapeutic and preventive actions.

### S089

#### Effectiveness of suicide prevention programs at emergencies and community mental health services

D. Palao Vidal[1,2]

Suicide is the leading cause of avoidable death worldwide (more than 58,000 deaths/year in Europe). People who attempts suicide are at high risk of further repetitions during the following year (12-30%). The lack of continuity of care following an acute suicide episode has been suggested as one of the major obstacles to effectively prevent suicide re-attempts. Secondary prevention programs have been developed to decrease the likelihood of a suicide re-attempt in patients at elevated risk. The European Alliance Against Depression (EAAD) project, showed a decrease in suicidal behavior. Since 2004, we have adapted the 4-level program of the EAAD in a catchment area of about 400.000 inhabitants A telephone post-attempt follow-up during 12 months and a stepped care model for depressed patients between Primary Care and Community Mental Health Centers were implemented. The program ensures the patient's engagement with the health care system, combining immediate face to face specialized care with telephone management, inclouding a clear action pathway to shorten the time frame between the participant's first contact with the health care system and delivery of specialized mental care. The evolution of the suicide and suicide attempts indexes were analyzed as main result of the program. Since 2014, this program has been generalized to the whole 7,5 M people Autonomous Community of Catalonia (Spain) with the name of Catalonia Suicide Risc Code (CSRC). We will discuss the difficulties in the implementation process in order to determine the barriers in the real effectiveness of this practical and efficient suicide prevention program.

Solid evidence is emerging signalling a significant weakening of psychiatric care during the "transition" process between Child and adolescent mental health care services (CAHMS) to adult mental health care services (AMHS). One of the major consequences of this fact is that care received by a high proportion of this vulnerable population is interrupted. This gap in their care pathway also happens before and after the transition itself, in a period considered critical. So far, the focus has been placed on studying and solving the problems derived from the passage between CAHMS to AMHS. But it is important to pay attention to the whole process including the previous phase before the age of transition Results of the CRECER study, an extensive study carried out in Madrid (Spain), reveal that more than 50% of individuals receiving care at CAHMS dropout the service just before the transition age (18 years old) without a medical discharge. This phenomenon could be considered as an actual black hole of care as the causes and the evolution of these individuals are unknown. Despite that, 10% of patients reappear again in AHMS being referred by primary care or emergency services, according to this study, The risk of drop-out of treatment around the age of the transition puts the continuity of care and the guarantee for adequate treatments at risk. Therefore, it should be considered as a relevant element in the design of mental health services for adolescents and young people and early care programs for all mental disorders.

### Perinatal depression: psychopathological characteristics and treatment strategies

### S103

#### Is pharmacotherapy safe in the perinatal period?

A. Wieck

Decisions about using psychotropic medications in pregnancy and lactation are among the most complex in clinical psychopharmacology, both for patients and their doctors. The uncertainty about the reproductive safety of these drugs contributes to the difficulty. Because of the absence of randomized controlled trials, data - coming from studies using other designs such as population or pregnancy registers and case-control comparisons - are difficult to interpret. This design issue is particularly important in the perinatal context, where pregnancy and child outcomes can be modulated by a host of other factors, such as substance misuse, socioeconomic disadvantage, physical illness, chronic stress and others. Nevertheless, the last few years have seen a growth of data on outcomes following the use of antidepressant medications in pregnancy and lactation. Most studies have examined the potential of these drugs for teratogenicity and indicate that the more commonly used antidepressants probably do not have a major teratogenic effect. Research is also growing on other outcomes, such as pregnancy and obstetric complications, neonatal Health and neurobehavioural development. In this presentation current research in these areas will be discussed.

**Conflict of interest:** No

### S105

#### Development and efficacy of a new psychoeducational intervention for peripartum depression

A. Fiorillo and M. Luciano*

Perinatal depression (PD) affects not only women but also their partner and children. The identification of PD is hampered by cultural, social and clinical factors. Therefore, an appropriate treatment according to international guidelines occurs only in a minority of cases. In case of mild to moderate forms of PD, cognitive behavioral therapy, interpersonal psychotherapy, and psychoeducation may be sufficient. Antidepressants are usually limited to severe forms of the disorder, since affected women prefer to avoid the use of medications for breast-feeding. Therefore, the early diagnosis of PD is essential in order to start treatment when depression is mild or moderate. In these cases, psychoeducational interventions reduce affective symptoms and the level of stress, and increase family functioning, and the maternal-newborn relationship. We carried out a study to develop and test the efficacy of a new psychoeducational family intervention in pregnant women with perinatal depression and their close relatives. This approach has been developed by a close collaboration between psychiatrists, gynaecologists, psychologists and other health professionals involved in the mother-baby units. The approach consists of six informative modules provided every 7-10 days on the clinical characteristics of the disorder, the early warning signs, the management of the relationship with the newborn and the family accommodation. Moreover, additional sessions on communication and problem solving skills are provided. 126 women and their partners have been involved in a randomized controlled trial, with TAU as control group. Our findings show that the experimental intervention is effective in the mild and moderate forms of PD.

### The ageing schizophrenia population: a new growing population with new challenges

### S118

#### Clinical evolution of schizophrenia in old age

A. Von Gunten

Older people with schizophrenia represent a significant part of those with serious and persistent mental illness. How schizophrenics live into older age and whether or not they are different from those with late-onset schizophrenia is still a matter of investigation, marginal though in a still neglected area. The clinical evolution over time, from adulthood into older age, including cognitive and medical comorbidities will be the focus of this presentation.

### S121

#### Long-term antipsychotic treatment in patients with schizophrenia: the gap between research and practice

M. Stuhec

Most trials including antipsychotics in schizophrenia exclude older adults on long-term antipsychotic treatment in both early and late-onset schizophrenia. Although there is some available data on this topic in this population within trials and meta-analyses, these patients are often excluded from existing treatment guidelines. On the other hand, long-term treatment can lead to serious problems including polypharmacy, drug-drug interactions, inappropriate prescribing and dosing, and treatment failure, which are especially important in older adults because of high polypharmacy and comorbidities. In this presentation we will present an overview of long-term antipsychotic treatment in schizophrenia in this population in relation to recommendations, clinical pharmacology and comorbidities, and show how to manage these issues in older adults who need long-term treatment with antipsychotics.

### Current challenges in the diagnosis and treatment of behavioural addictions

### S123

#### Compulsive buying: is there enough evidences to recognize it as a disorder?

L. Claes

In 2018, an international group under supervision of Prof. dr. Astrid Müller wrote a paper in which they stated that it is really time to recognize Compulsive Buying (CB) as a separate mental health condition (Müller et al., 2018, CNS Spectrums, 1-6). My talk will be inspired by this statement paper of which I was a co-author. CB is often defined by the excessive preoccupations with buying/shopping, that are experienced as irresistible, and recurrent maladaptive buying/shopping excesses that lead to marked distress and impairments. A meta-analysis indicated a prevalence rate of CB of 5%, which is increasing in developing societies .The majority of studies found that CB is higher among women, certainly in treatment-seeking samples. Comorbidity rates with CB are high, with mood disorders, hoarding disorder, eating disorders and gambling and substance use disorders as well as personality disorders. There are some commonalities between CB and obsessive-compulsive disorders, impulse control disorder, substance-use disorders and gambling disorder, the latter suggesting that CB might be considered as a behavioral addiction. Considering recent research findings, the prevalence of CB, the interference with daily functioning and the number of treatment seeking people, it is really time to recognize CB as a separate mental health condition.

### New ideas and technologies in the treatment of anxiety and psychotic disorders

#### S128

#### Cognitive behavioural therapy augmented with virtual reality exposure for treatment of social anxiety: preliminary results from a qualitative pilot

B. Thorup* and P. Bang

**Background:** Several randomized controlled trials examining the efficacy of Virtual Reality Exposure (VRE) for treating Social Anxiety Disorder (SAD) have found similar effects compared with well-established therapeutic treatment types (Anderson et al., 2013; Bouchard et al., 2017; Klinger and Bouchard, 2005b; Kampmann et al., 2016). However, to date, no study has investigated the use of VRE in a pragmatic clinicial outpatient group CBT setting.
**Aims & Method:** The present study is a qualitative evaluation of a newly developed group cognitive behavioral therapy (CBT) VRE treatment fitted to the Danish psychiatric system. The aim of this study is to evaluate how the challenges of implementing VRE into a 'living and breathing' pragmatic clinical group CBT setting are met and if needed to gather the information necessary to implement meaningful changes to the intervention. Information was gatherered through semi-structured interviews of 11 patients who have undergone group CBT with VRE treatment, as well as interviews from 4 therapists who performed the treatment. The interviews were transcribed and analysed using Thematic Analysis.
**Results:** The analysis resulted in the following themes: "Technical challenges", "VRE as meaningful in multiple ways", "Positive side-effects" and "The duality of VRE exposure in a group setting".
**Conclusion:** The themes were discussed with the people involved in the treatment and lead to several changes in the implementation: Revisions in the the amount of VRE delivered, revisions in the way of deliverying VRE and revisions in the practical circumstances of the VR technology (Storage, charging and so on...).

### Treating anxiety, depression and bipolar disorders: aiming at the amygdala

#### S130

#### Anxiety, mindfulness and the amygdala

C. Piguet

In the last two decades, given a growing interest for non-pharmacological approaches to anxiety disorders, mindfulness-based interventions (MBI) have received serious attention. A methodologically rigorous meta-analysis of RCT confirms a substantial effect on anxiety, depression and pain (Goyal et al., 2014). Recent meta-analyses reported a positive effect of MBI on stress, anxiety, and depression in adolescents as well (Kallapiran et al., 2015, Zoogman et al., 2015). At the brain level, reviews report that adult subjects seem to show an increased activity in the anterior (ACC) and posterior (PCC) cingulate cortex and prefrontal cortex, and a reduction of activity induced by emotional stimuli, including in the amygdala (Fox et al., 2016; Gotink et al., 2016). Increased activity in the amygdala has been repeatedly associated with anxiety disorders (Etkin & Wager, 2007; Fonzo et al., 2015). On the other hand, increased stress reactivity during adolescence has been associated with vulnerability for various stress-related disorders including full-blown anxiety disorders (Monroe and Harkness, 2005). Since adolescence is a critical period for the shaping of the cortico-limbic emotion regulation networks (Blakemore, 2019; Tottenham & Galván, 2016), we can expect adolescents in particular to show increased amygdala reactivity correlating with anxiety during stress tasks. Therefore, MBI could be a potent therapeutic tool to decrease anxiety-induced limbic hyperactivity, in adults but also in adolescents, making it a current object of investigation in early intervention studies.

**Conflict of interest:** No

#### S131

#### Role of insula to amygdala projection neurons in anxiety- and valence-related behaviors

A. Beyeler

Most pharmacological treatments of anxiety disorders are targeting the serotonin system. Although the insular cortex (IC) has been described to be overactive in patients with anxiety disorders, the implication of specific neural populations and serotonin receptors located within this region, in the control of anxiety remains unknown. Using viral anterograde tracing we found that the anterior and posterior insula neurons, preferentially project to the basolateral (BLA) and central amygdala (CeA) respectively. Optogenetically assisted circuit mapping allowed us to demonstrate a monosynaptic connection of the insula neurons to BLA glutamatergic neurons and CeA GABAergic neurons. We also identified that 80% of IC-BLA and IC-CeA projectors express the serotonin 1A or 2A receptors, while only one third of GABAergic interneurons express these receptors. Using fiber photometry, we found that projection neurons of the anterior, but not posterior insula are more active in anxiogenic spaces. Interestingly, recordings of IC-BLA neurons revealed that the IC-BLA populations is also more active in pro-anxiety environments. Finally, we found that optogenetic activation of IC-BLA neurons induce real time place preference while activation of IC-CeA projectors induces real time place aversion. Altogether, our findings revealed a new role of the anterior insular cortex and IC-BLA projection neurons in anxiety-related behaviors. We acknowledge the support of the Région Nouvelle-Aquitaine, the INSERM-Avenir program of the French NIH (INSERM) and the Fondation NRJ-Institut-de-France to the Beyeler Lab, and of the Brain and Behavior Research Foundation NARSAD young investigator grant to Anna Beyeler.

### S133

#### Fmri based neurofeedback of the amygdala in bipolar disorder

P. Favre[1,2]

Decades of research support the role of amygdala in emotional processing. The amygdala is strongly involved in the generation and identification of emotions. Interactions between amygdala and prefrontal cortex are central to the modulation of the affective response. In Bipolar Disorder (BD), there is evidence for amygdala overactivation and a lack of prefrontal activation during emotional paradigms (Chen et al., 2011), which are likely to reflect abnormal emotion regulation in patients with BD (Kanske et al., 2015). Interestingly, studies showed a modulation of amygdala's activation after a pharmacological treatment (Strakowski et al., 2016). This led to the idea that normalization (i.e., downregulation) of amygdala activity may be associated with attenuated emotional-hyper-reactivity in patients with BD, and possibly with clinical improvements for instance regarding persistent sadness (Posse et al., 2003). Targeting amygdala's activity is therefore a promising therapeutic strategy to relieve thymic symptoms in BD. The real-time fMRI neurofeedback (NFB) is an innovative technique that allows to record the signal from a given brain region and to display it back in real-time to the participant. With this feedback, subjects can learn to control the activity of selected brain areas (Weiskopf et al., 2004). Amygdala-based NFB training has already shown positive effects on the management of mood and affective symptoms in major depressive disorder (Young et al., 2017) and borderline personality disorder (Paret et al., 2016). Therefore, its application for the treatment of BD is very promising.

### Can quality assurance of psychiatric services reduce stigma and discrimination?

### S139

#### Quality assurance: systemic processes and information to obtain consent

T. Akiyama

Quality assurance in medicine includes both systemic processes and the provision of appropriate information to obtain consent for clinical procedures. Systemic processes consist of 5 components. 1. Policy—the facility should establish policies that cover essential areas such as governance, patients' rights, care process, education for patients and care-givers, high-risk procedures, use of laboratory and radiology tests, use of medication, oversight for outsider service providers comprehensively. 2. Education and training—the facility should provide appropriate opportunities for education and training for the staff to learn and understand the policies. 3. Monitoring—The facility should monitor how appropriately the policies are implemented. 4. PDCA cycle—When the facility detects a need to improve, it should implement Plan-Do-Check-Action (PDCA) cycle. The effects of the PDCA cycle should be monitored with an indicator. 5. Communication—The facility should convey information about how these quality assurance efforts are done to the patients and the care-givers. The information to obtain consent should include 1. Diagnosis and symptoms 2. Treatment plan 3. Content of procedure 4. Expected effects 5. Possible adverse effects 6. Care to adverse effects 7. Comparison with substitute procedures 8. Prediction when no procedures are taken If psychiatry succeeds in establishing the systemic processes and ensuing to provide appropriate information to obtain consent, patients will feel that the treatment is provided appropriately and respectfully. This should decrease the risk of self-stigma. The psychiatrists can provide treatment in the level of other Medicine end reduce stigma through clinical procedures.

### S141

#### The contribution of quality medicine to overcoming stigma and discrimination of people with mental illness

W. Gaebel

People with mental illness experience different types of stigma and discrimination (Gaebel, Rössler, & Sartorius, 2017). These experiences present a severe burden for those affected and lead to reduced self-esteem, social participation, and overall quality of life. Additionally, stigma and discrimination are barriers to the utilization of mental healthcare and lower help-seeking behavior of those affected by mental illness. Thus, various stakeholders have proposed recommendations to reduce the stigma of mental illness. Some of these recommendations focus on structural aspects and the quality of healthcare systems. Quality is defined as a complex construct with structure-, process-, and/or outcome-oriented features on different levels of observation (macro-, meso-, micro-level) and with different perspectives (physicians, patient, providers, etc.). It describes the degree to which health services increase the likelihood of desired health outcomes and are consistent with current professional knowledge. Promising approaches in the context of quality medicine that may jointly contribute to reducing stigma and discrimination in mental illness include evidence-based quality management, value based care (i.e., a focus on relevant health outcomes relative to the costs of delivering the outcomes), and "choosing wisely" (i.e., an initiative to improve shared decision-making based on evidence). In this presentation, contributions of concepts, methods, practical approaches of quality Medicine end available evidence to overcoming stigma and discrimination of people with mental illness will be elaborated. Gaebel, W., Rössler, W., & Sartorius, N. (Eds.). (2017). The Stigma of Mental Illness-End of the Story? Heidelberg, Germany: Springer.

### Are psychodelics useful in the treatment of mental disorders?

#### S142

#### Psychedelics revisited: therapeutic potential of pharmacological outcasts

P. Mohr[1,2]

Psychedelics are the least clinically utilized class of psychotropic drugs, with a long history of use in traditional Medicine end religious rituals. The group primarily consists of serotoninergic 5-HT2a agonists, serotonin releasers, glutamatergic NMDA antagonists, and cannabinoid agonists. Despite (or rather due to) their potent pharmacological action, inducing thought and perceptual changes, altered states of consciousness, psychedelic drugs are illicit, vilified, and feared. More recently, there has been a kindled interest in exploring their therapeutic utilization, beyond psychotherapy. Functional studies in healthy subjects help us to elucidate neurobiological changes in the brain induced by these compounds; clinical trials in patients yielded encouraging results. In addition to robust evidence of antidepressive and antisuicidal effects of ketamine, other psychedelic agents showed a promising potential in treatment of depression, PTSD, OCD, addiction, anxiety and fear associated with terminal illness. Current data strongly suggest that efficacy and safety of psychedelic drugs need to be further investigated and their place in clinical practice reconsidered, based on the study results.

#### S143

#### Functional changes induced by psychodelic drugs

K. Preller

The use of psychedelics in scientific and experimental clinical contexts has triggered renewed interest in the mechanism of action of psychedelics. However, their systems-level neurobiology and neuropharmacology remains sparsely investigated in humans. This talk will present randomized placebo-controlled pharmacological neuroimaging studies conducted with the prototypical psychedelics LSD and psilocybin. Here, we leverage newly developed data-driven and computational data analysis methods revealing the impact of these substances on brain connectivity. To further investigate the pharmacology underlying psychedelic-induced alterations in functional connectivity, compare those effects to cortical receptor gene expression maps. We show that 1) psilocybin and LSD reduce associative, but concurrently increase sensory brain-wide connectivity; 2) LSD alters directed (effective) connectivity between cortico–striato–thalamo-cortical regions in accordance with the thalamic filter model; 3) LSD and psilocybin induced changes correlate time-dependently with spatial gene expression patterns in particular of the 5-HTR2A, 4) subjective and neural effects of LSD can be blocked with the serotonin 2A receptor agonist ketanserin. Together, these results advance our mechanistic understanding of the action of psychedelics. This is important for the development of new pharmacological therapeutics and also increases our understanding of the mechanisms underlying the potential clinical efficacy of psychedelics.

### Current picture and future directions of child and adolescent psychiatry training in europe

#### S146

#### Cap in gap: a look at the quality of cap training for adult psychiatry trainees

A. Seker

Child and Adolescent Psychiatry (CAP) is becoming more prominent for the last few decades, however, the shortage of specialists is a global issue. This shortage brings about a lot of struggles, including difficulties contacting a CAP specialist both for patients and medical students/trainees. CAP is not an individual specialty in many countries, including European ones, and adult psychiatrists end up assessing and treating CAP patients in routine practice. Even when it's not the case, a proper CAP exposure is very important during training for every psychiatric branch. European Federation of Psychiatric Trainees (EFPT) CAP Working Group is acknowledging the need for a quality CAP rotation during general psychiatry training. 'CAP in GAP' survey has been created by the EFPT CAP Working Group members to explore the CAP rotation circumstances in Europe. This survey looks into many different factors, including the mandatory nature, duration, content and relevance of CAP rotations while questioning factors influencing the satisfaction of trainees. In this presentation, the results of the 'CAP in GAP' survey are discussed with a particular focus on different training systems where CAP is an individual specialty, a sub-branch or an unofficial specialty interest included in general psychiatry. The implications of different CAP training rotation curricula for everyday practice is also considered.

#### S148

#### Current challenges and opportunities in cap training throughout europe: a trainee perspective

R. Mazaliauskienė[1] and S. Gnanavel[2*]

It is well recognised that Child and adolescent psychiatry (CAP) training for psychiatrists is sub-optimal, across the globe, including in Europe. Lack of standardisation, benchmarks and lack of focus on practical competencies results in lacunae in CAP training. While there is some literature summarising European perspectives in CAP training in general, there is little understanding from trainee's perspective. I will be presenting findings from our recently concluded study on "Child and adolescent psychiatry training curriculum: A global trainee's perspective" pertinent to Europe. This would summarise unmet needs in clinical, academic and research training from CAP trainees perspective. It is encouraging to note the efforts of the European Union of Medical Specialists Section on Child and Adolescent Psychiatry (UEMS-CAP) and EFPT working to create a common European standard in CAP training.

### Improving lifestyle behaviours in people with severe mental disorders

#### S151

#### Reducing mortality gap in schizophrenia: healthy lifestyles and pharmacological treatment

M. Pompili

Reduction in life expectancy among patients with schizophrenia is a major mental health issue worldwide. Such reduction has been reported to be even more alarming when youths are compared with middle-aged patients. The loss of life is especially due to suicide and cardiovascular disease. Such latter cause is correlated with lifestyle factors,antipsychotic medication,inadequate screening for risk factors and inadequate intervention contribute to morbidity. Lifestyle factors associated with schizophrenia can increase the risk of diabetes. There are warnings of the risk of hyperglycemia/diabetes for most SGAs; mechanisms may include weight gain and insulin resistance; such difference in risk is very similar to that seen with weight gain. Achieving a balance between adequate symptom control and a variety of other unmet needs is becoming a focal point of decision making in psychiatric care. Evidence supports the notion that the risk increases immediately after stopping antipsychotics and remains high over time; on the other hand, maintaining antipsychotic treatment is associated with a reduced risk of relapse, fewer hospitalizations, and a better quality of life compared with a placebo or no treatment. The number of hospitalizations is a central risk factor for suicide as each admission is indicative of poor outcomes, a painful awareness of ineffective treatment, and a fear of further mental disintegration. Treatments with some of the LAIs available could reduce both mortality and hospitalizations. Relapse may increase immediately after stopping antipsychotics and remains high over time; while maintaining antipsychotic treatment is associated with reduced morbidity, mortality and better quality of life.
**Disclosure:** Advisory boards: Janssen, Ferrer, Lectures: Angelini, Otsuka, Lundbeck, FB Health, Allergan

#### S152

#### Can psychosocial treatment reduce antipsychotic-induced metabolic disturbances in patients with first episode psychosis?

Weight gain and metabolic abnormalities associated with antipsychotic medication are major challenges in the treatment of patients with first episode psychosis. Unfortunately, current strategies for preventing or reducing these side effects are not successful. This study aims to analyse the effects of a multimodal treatment program for first episode psychosis on metabolic changes during the first 18th months of treatment. 71 patients who were treated at the Zagreb University Hospital Centre for first acute psychotic episode from 2016 until 2018, were included in the study. After 3-4-week long hospital treatment, patients attended either the day hospital program or treatment as usual (TAU) consisting of short outpatients visit once a month. We monitored baseline and final body weight, blood glucose, lipids and cholesterol as primary outcome measures and psychopathology and global level of functioning as secondary outcome measures. After the adjustment for age, gender and baseline measures, the type of treatment was not significantly associated with any of the primary outcome measures and both types of treatment were relatively ineffective in counteracting medication-induced metabolic abnormalities and weight gain. Patients' psychopathology measures significantly decreased, and their functional level significantly increased at month 18th compared to the baseline values in both groups. Thus, even though both treatment options reduced psychopathology and helped restoring the patients' level of functioning over the first 18 months of treatment, it is possible that over a longer period the negative effect of medication associated metabolic side effects may surpass the beneficial effect on the psychiatric disorder.
**Disclosure:** This research was supported by the grant of the Croatian Science Foundation No UIP-2014-09-1245 Biomarkers in schizophrenia-integration of complementary methods in longitudinal follow up of first episode psychosis patients. The funding source was not invo.

#### S153

#### The role of psycho-educational interventions in improving physical health of people with severe mental illness

A. Fiorillo and G. Sampogna*

People with severe mental disorders (SMD) have a reduced life expectancy of about 10-20 years compared with the general population. In order to reduce this mortality gap, several strategies have been implemented at population and individual levels. Among the latter, ad-hoc psychosocial interventions have been developed with the aim to improve unhealthy lifestyle behaviours, such as tobacco smoking, diet, physical exercise, drug adherence, and risky sexual activities, which are partially responsible of this premature mortality. Different psychoeducational interventions have been developed, differing for several aspects, including format, setting, duration, and involvement of health professionals (such as dieticians or trainers for physical activities). Most of these approaches include motivational, educational and problem-solving components. Their efficacy in randomized controlled trials has been documented, but their effectiveness on the long-term has not been proved yet. Furthermore, the dissemination of these approaches on a large scale is not satisfying, particularly in low- and middle-income countries, where mortality rates for infectious diseases are higher and availability of healthcare resources is lower.

### Internet interventions with CBT

#### S154

#### Scientific basis for ICBT

T. Berger

During the last 20 years, many internet-delivered psychological treatments have been developed and tested for a wide range of clinical problems. Therapist-guided internet treatments are effective in controlled trials on mood and anxiety disorder, but also other conditions and several health problems. While most research has been on disorder-specific cognitive-behavioral treatments, there are also studies on transdiagnostic and tailored approaches addressing common elements of several disorders and comorbid problems. Controlled trials indicate that guided internet-treatments can be as effective as face-to-face treatments, that they lead to sustained improvements, work in clinically representative conditions, and are cost-effective. Less is known about moderators and mediators of change. Psychotherapists and psychiatrists in the future will likely use internet treatments as complements to face-to-face therapy. This presentation will give an overview of the current state of research in ICBT.

### Non-Invasive brain stimulation for late life depression: challenges and perspectives

#### S160

#### NIBS for TRD in the elderly: dealing with comorbidities

B. Calvet

Electroconvulsive therapy (ECT) and Repetitive transcranial magnetic stimulation (rTMS) differ in mechanism, tolerability, and acceptability by patients and may be best understood as complementary rather than competing techniques. ECT remains psychiatry's gold standard treatment for severe mental illness, including mood disorders and psychosis. It can be used in a variety of patient populations and has a particularly salient role in the treatment of geriatric patients with depression. Despite its proven efficacy and safety, shortcomings of ECT include its adverse effect profile (mainly temporary cognitive impairment).
On the other hand, rTMS has a high degree of tolerability and safety among older elderly patients, leading to conclude that elderly people with Treatment-resistant depression should not be excluded in clinical trials or practice. However, this technique would be less effective in the elderly. In fact, there have been remarkably few studies investigating this age-effect hypothesis directly, and it is difficult to draw any meaningful conclusions due to marked methodological variability in terms of coil placement, "dosing " (e.g., stimulation frequency and intensity, number of pulses), and treatment duration. Some studies show that older patients with TRD have comparable outcomes to younger patients and demonstrate that age alone should not be considered as a contraindication or poor prognostic indicator to TMS therapy.
This oral communication will identify the different comorbidities of depression to be managed as well as stimulation parameters to be applied to optimize the use of NBIS in depression in the elderly.

#### S166

#### Virtual reality in primary prevention: bullying in the classroom

M. Barreda[1*], M. Serra-Blasco[2], M. Cano-Català[2], A. Pereda-Baños[3], X. Goldberg[2] and N. Cardoner Álvarez[4]

**Introduction:** Working on the development of pupil's cognitive-emotional skills has been shown to be a key factor for the success of anti-bullying interventions in primary and secondary school. Virtual Reality (VR) technologies, which have been claimed to be a powerful "empathy machine", could have great potential in this regard.
**Objectives:** To examine whether immersion on a first-person VR experience representing a situation of school bullying impacts empathy and attitudes towards victims in secondary school students.
**Methods:** Students participated in a co-creation task for creating the VR scenes, which were pre-tested with a different sample of pupils. Self-reported measures of the experience and attitudes towards bullying were collected, together with psychophysiological measures during the viewing.

**Results:** Preliminary results suggest that the immersive experience elicits realistic responses in participants, enhances emotional reactions towards the situation, and might contribute to more pro-social attitudes.
**Conclusions:** The results encourage further research exploring the specific aspects of VR interventions in school bullying prevention programs and their impact on behavior.

### S169

### When digital technologies met neuroimaging: implications for clinical psychiatry

In recent years, there has been an unprecedented progress in the identification and characterization of the neurobiological correlates of different disorders of mental health. The use of non-invasive neuroimaging techniques, such as Magnetic Resonance Imaging (MRI), has allowed performing such assessments in real patients, and, in consequence, developing neurobiological models directly accounting for patients' symptoms, which may purportedly predict relevant outcomes from a clinical standpoint, such as disorder's course or treatment response. Obviously, these developments rapidly attracted the interest of clinicians and funding entities, but early promises of clinical translation have been toned down because, although progresses have been made to describe the structural and functional bases of mental health disorders at the group level, it has been difficult, up to now, to develop robust methods to make predictions at the individual level, which is essential for a real clinical translation. In this context, new methods of analysis based on digital technologies approaches have been increasingly applied to neuroimaging data. These methods, including machine learning, deep learning or artificial intelligence applications, can process large amounts of data to make predictions about the most likely diagnose, evolution or response to a particular treatment of an individual. Nevertheless, it is yet not clear how neuroimaging information should be integrated with other kind of data (from omics to different types of behavioral and contextual data) to make such predictions. Moreover, there is a need for developing libraries of normative data to make clinically meaningful predictions.

## News and mechanism of action (MOA) of ECT in depression

### S178

### The truth about electroconvulsive therapy: a new era of knowledge. clinical predictors of therapeutic response to electroconvulsive therapy

Electroconvulsive therapy (ECT) is considered the most effective short-term treatment for patients with severe or treatment-resistant depression. However, approximately one-third of the individuals with depression do not respond to electroconvulsive therapy (UK ECT Review Group, 2003; APA, 2010). Reliable predictors of ECT response would be useful for patient selection, but have not been demonstrated definitively. There has recently been a growing interest on clinical predictors affecting ECT outcome for depressed patients, including sex, psychotic features, medication resistance, melancholic features, polarity of mood disorder, age, duration of the current depressive episode and duration of illness (Bloch et al., 2005; Petrides et al., 2001; Heijnen et al., 2010; Fink et al., 2007; Sienaert et al., 2009; Medda et al., 2014; Altamura et al., 2015). Recent meta-analytic evidence shows that longer depressive episodes and medication failure at baseline are robust predictors of poor response to ECT (Haq et al. 2015). Moreover, the presence of psychotic features and older age are predictors of ECT remission and response. The severity of depression predicts response, but not remission (Van Diermen et al., 2018). Patients' expectations prior to the treatment have been taken into consideration in few studies. ECT effectiveness does neither seem to be subject to a strong placebo effect nor do patients with lower expectation of ECT effectiveness respond better to treatment (Krech et al., 2017). More research on both biological and clinical predictors is needed to further evaluate the position of ECT in treatment protocols for major depression.

### S180

### Mechanism of action of electroconvulsive therapy: a multimodal neuroimaging model

Electroconvulsive therapy (ECT) is an effective alternative treatment for patients with treatment-resistant depression (TRD). Although its mechanism of action remains poorly understood, multimodal neuroimaging data (i.e., resting-state functional connectivity (FC), brain morphometry and brain spectroscopy) could provide a better understanding of the neurobiological bases of ECT in patients with TRD. This presentation will review current neuroimaging research on the mode of action of ECT in patients with TRD. First, I will discuss how a complex interaction between early and late limbic-prefrontal ECT-induced FC changes have an impact on clinical improvement of patients with TRD. Second, I will show how different neuroplastic (i.e., angiogenesis, gliogenesis and synaptogenesis) and neuroinflammatory changes may be induced by ECT and explain its mechanism of action. Finally, I will integrate all these multimodal neuroimaging findings in a single model of ECT mechanism of action in patients with TRD.

### S181

#### Gemric: the global ect-mri research collaboration

L. Oltedal[1,2]

The neurobiological effects of ECT have been subject to investigations by use of Magnetic Resonance Imaging (MRI) for two decades. These studies have significantly advanced our knowledge of brain changes following ECT, however the results have also been inconsistent, particularly with respect to the relationship between MRI neurobiological effects and therapeutic outcomes. Since the establishment of the Global ECT-MRI Research Collaboration (GEMRIC) in June 2015, research groups around the world has teamed up and share data in order to increase our knowledge. Currently, 20 sites contribute in this effort, and our common dataset includes > 700 participants (including controls). This large dataset has provided the statistical power needed to characterize whole-brain structural and functional brain changes following ECT. The collaboration has so far published five papers, and a number of mega-analyses which have been approved by the GEMRIC board are currently ongoing. So far, the collaboration has investigated ECT effects on the hippocampus as well as on whole-brain gray matter. Moreover, we have documented how the electrode position and the electrical field distribution relates to volumetric brain changes. The current status, results and future prospects of GEMRIC will be discussed.

### Adult offenders and child victims of sexual abuse: an update for psychiatrists

### S182

#### General introduction to sex offending emphasising prevalence, diagnostics and risk assessment and risk management

K. Goethals[1,2]

In this paper sex offending will be addressed, especially with regard to the prevalence, diagnostics and risk assesment tools and risk management. Next, patients with paraphilic disorders and/or hypersexuality will be considered. Finally recommendations for clinical practice will be made.

### S184

#### Consequences of child abuse and neglect in adulthood

P. Plener

Adverse childhood experiences are still commonly found in a high number of children and adolescents. The risk for developing mental health disorders such as (complex) posttraumatic stress disorder (PTSD), depression or others is especially increased in survivors of interpersonal traumatic experiences. Recent years have seen an increased understanding of underlying neurobiological mechanisms, especially those involved in the central stress response, epigenetic changes as well as alterations in the immune response. Therefore, findings from mental and somatic health concerning and increased risk for the development of chronic disorders are being bridged through a joint understanding. There is a growing body of literature suggesting that the risk for several somatic disorders is increased in survivors of adverse childhood experiences. The presentation will focus on connections between adverse experiences, neurobiology, mental-, as well as somatic health.

### Identifying and targeting suicide risk factors in youth

### S187

#### Protective and risk-conferring factors for suicidality in youth in europe

M. Sarchiapone[1,2]

Suicide represents the third leading cause of death among adolescents aged 15-19 years. Suicide is a very complex, multicausal phenomenon determined by the action of several bio-psycho-social factors. The stress-diathesis model was proposed to explain this complex interactions, suggesting that the vulnerability to suicide is influenced by a variety of predisposing (distal) risk factors on which stressful life events and other potentiating (proximal) factors may act as triggers. Nevertheless, the influence of these risk factors can be mitigated by the action of protective factors. Identified risk and protective factors for suicidality among youngsters are similar to those found among adults, even if they present some specificities.
This presentation will discuss findings from the EU funded projects SEYLE (Saving and Empowering Young Lives in Europe) and WE-STAY (Working in Europe to Stop Truancy Among Youth), as well as data from a large epidemiological study funded by UNICEF Kazakhstan. Specific risk and protective factors for youth suicidal ideation and behaviours will be discussed in the framework of the diathesis-stress model integrating biological, psychological, social and situational factors.
A deeper understanding of risk and protective factors may help to develop more effective suicide prevention interventions really able to counteract the influences of the former and strengthen the positive effects of the latter.
**Disclosure:** The SEYLE and WE-STAY projects were funded by the EU under the Seventh Framework Programme for Research (FP7). I was a consultant for UNICEF Kazakhstan in the study "Adolescent Mental Health Promotion and Suicide Prevention Programme in Kazakhstan".

**Keywords:** Suicide; adolescents

### S188

### Perspectives of modern society in youth suicide prevention

M. Pompili

Suicide is a leading cause of death for children, adolescents, and young adults. There is evidence to support that the suicide rate for those aged 15 to 29 years has tripled since 1950, and suicide is now the second or third leading cause of death in this age group. The role of caregivers and schools (as well as colleges and universities) is important in the assessment, management, and Prevention of suicidal behavior in children and youth. Recognition of risk factors is of paramount importance for prevention. Psychosocial factors significantly increase the risk of suicide in children and adolescents, regardless of any psychiatric disorder present. The major psychosocial risks for adolescents have been poor communication with the father, a father with a history of police problems, a family history of suicidal behavior, disciplinary crises, recent losses (for boys), and school or work problems. An altered family structure has previously been reported as more related to the risk of involving adolescents in suicide attempts or other risky behaviors. Suicide is now conceptualized with neurodevelopmental origins. Children and young adolescents frequently show emotional and behavioral disturbances associated with irritability during the period preceding the suicidal act. Early childhood environmental risk factors should be considered in the understanding of how individuals may become vulnerable to suicide risk. Pro-suicide websites are also major issues. The association between bullying, harassment, victimization, and irritability may contribute to suicide risk in this age group.

### S189

### Treating and preventing suicidality in youth: assessing the evidence for efficacy

D. Wasserman

Suicide in young people is in many countries' worldwide the leading cause of death. In this age span approximately 20-30 times as many suicide attempts occur. Suicide attempt is one of the most important predictors of future suicide and therefore deserves vigorous suicide preventive strategies. The latest data on pharmacological and psychological treatment as well as the results of mental health promotion and suicide preventive programs in schools will be presented along with cost effective strategies.

## More treatment but not less depression: the paradox of over- and under-diagnosis in depression

### S193

### More treatment but not less depression: reasons and remedies

J. Ormel

Treatment of depression has increased sharply since the 1980s. With more prescriptions of antidepressants and psychological treatments, more depressed people are receiving help. This should have resulted in a decrease in depression's point-prevalence. Remarkably, though, epidemiological studies do not find a drop in prevalence. Why might this be? It is a key point to reconcile, for this paradox raises fundamental questions regarding present-day treatments. We evaluate five explanations for this "treatment-prevalence paradox" (TPP). The first two hypotheses share the assumption that there the point-prevalence has dropped but that this drop has been masked by an offsetting 1) increased incidence of depression due to an increase of risk factors and/or 2) growing tendency to classify and diagnose distress as major depression. Alternatively, hypotheses 3-5 share the assumption that there has not been a treatment-driven prevalence reduction, but rather that treatment does not lessen the population prevalence. This could come about because 3) treatments continue to be inadequate for many patients; 4) RCTs overestimate treatment efficacies; 5) the efficacy-effectiveness gap, i.e. treatments do not work as well in real-world settings. We conclude 1) that most explanations contribute to the explanation of the paradox (except the incidence increase) and 2) that Prevention of incidence and recurrence seems essential to achieve less depression. Unfortunately, so far preventive efforts have not succeeded in reducing depression burden either. This, we argue, arises from a lack of socially-embedded, structurally funded prevention, legal consolidation, and the difficulty of reducing the impact of major determinants.

**Conflict of interest:** No


Article ID: S005
Subjects: Abstract, Cognitive Impairment across the Schizophrenia Spectrum: Focus on Assessment and Treatment

The impact of cognitive and social cognitive impairment on real-life functioning in subjects with schizophrenia

Sachs G. 1 *
Erfurth A. 2
1 Medical University of Vienna , Department of Psychiatry and Psychotherapy, Vienna , Austria
2 Krankenhaus Hietzing, 1st Department of Psychiatry and Psychotherapeutic Medicine , Vienna , Austria
* Corresponding author.
Keywords: neurocognition, Social Cognition, functional outcome, schizophrenia

Conflict of interest:

No

Article ID: S008

New Developments of Cognitive Remediation in Schizophrenia

Wykes T.
Institute of Psychiatry , Psychology and Neuroscience, King’s College London, Department of Psychology, London , United Kingdom
Keywords: cognitive remediation, schizophrenia, psychosocial rehabilition, recovery

Conflict of interest:

No

Article ID: S009
Subjects: Abstract, Transdiagnostic Perspective of ADHD Among Different Mental Disorders

ADHD and Obesity: Dopaminergic Signaling as Biological Link

Roth Mota N. 1 *
Poelmans G. 1
Klein M. 1
Torrico B. 2
Fernàndez-Castillo N. 2
Cormand B. 2
Reif A. 3
Franke B. 1
Arias Vásquez A. 1
1 Radboud university medical center , Department of Human Genetics, Nijmegen , Netherlands ,
2 Universitat de Barcelona, Departament De Genètica, Microbiologia I Estadística , Barcelona , Spain ,
3 University Hospital Frankfurt , Department of Psychiatry, Psychosomatic Medicine end Psychotherapy, Frankfurt Am Main, Germany
* Corresponding author.
Keywords: obesity, comorbidity, GWAs, ADHD

Conflict of interest:

No

Article ID: S012

Investigating the effects of physical activity on positive and negative affect in the everyday life of patients with adhd – a mobile health approach

Koch E. 1 *
Freitag C. 2
Mayer J. 2
Reif A. 3
Grimm O. 4
Ramos-Quiroga J.A. 5
Ibáñez P. 5
Palacio J. 5
Kuntsi J. 6
Pawley A. 6
Buitelaar J. 7
Bergsma D. 7
Ebner-Priemer U. 1
1 Karlsruhe Institute of Technology, Mental Mhealth Lab., Institute of Sports And Sports Science , Karlsruhe , Germany
2 University Hospital Frankfurt , Goethe University, Department of Child and Adolescent Psychiatry, Psychosomatics, And Psychotherapy, Frankfurt am Main , Germany
3 University Hospital Frankfurt , Department of Psychiatry, Psychosomatic Medicine end Psychotherapy, Frankfurt am Main , Germany
4 University Hospital Frankfurt , Department of Psychiatry, Psychosomatic Medicine end Psychotherapy, Frankfurt Am Main, Germany
5 Hospital Universitari Vall d'Hebron. Universitat Autònoma de Barcelona, Department of Psychiatry , Barcelona , Spain
6 King’s College London, Mrc Centre For Social Genetic & Developmental Psychiatry , London , United Kingdom
7 Radboud University Nijmegen Medical Centre , Department of Cognitive Neuroscience, Nijmegen , Netherlands
* Corresponding author.
Keywords: Ambulatory Assessment, Physical Activity, affect, ADHD

Conflict of interest:

No

Article ID: S023
Subjects: Abstract; Understanding the Variation of the Human Brain: Genes, Sex, Environment and Psychopathology

Does fetal testosterone differently influence male and female brain development?

Ruigrok A. 1 *
Holt R. 1
Dooley N. 1
Lombardo M. 2
Auyeung B. 3
Baron-Cohen S. 1
1 University of Cambridge , Autism Research Centre, Department of Psychiatry, Cambridge , United Kingdom
2 @UniTn, Istituto Italiano di Tecnologia, Laboratory For Autism And Neurodevelopmental Disorders, Center For Neuroscience And Cognitive Systems, Roverto , Italy
3 The University of Edinburgh , Department of Psychology The School of Philosophy, Psychology And Language Sciences, Edinburgh , United Kingdom
* Corresponding author.
Keywords: brain development, sex steroid hormones, fetal testosterone, sex differences

Conflict of interest:

No

Article ID: S032
Subjects: Abstract, A New Model to Manage Difficult to Treat Depression

Is bipolar depression a different type of depression?

Fiorillo A.
University of Campania "L. Vanvitelli" , Department of Psychiatry, Naples , Italy
Keywords: mixed state, controversies, Bipolar depression, Mood disorders

Conflict of interest:

No

Article ID: S036
Subjects: Abstract, Etiopathogenesis of Suicidal Behaviour and Major Depression: Novel Approaches

Genomics of suicidal behaviour

Courtet P.
University of Montpellier. , Department of Emergency Psychiatry & Acute Care, Montpellier , France
Keywords: Suicidal behaviour, opioid, treatment emergent suicidal ideation

Conflict of interest:

No

Article ID: S041
Subjects: Abstract, E-Mental Health across the Lifespan

Mindfulness and virtual reality in adults with adhd: results of a randomised, controlled clinical trial

Ramos-Quiroga J.A.
Hospital Universitari Vall d'Hebron. Universitat Autònoma de Barcelona, Department of Psychiatry . Cibersam, Barcelona, Spain
Keywords: ADHD, ADULTS, VIRTUAL REALITY, MINDFULNESS

Article ID: S042

The super brains app for adhd

Kooij S.
Amsterdam Medical University Center , location VUmc, Psychiatry, Amsterdam , Netherlands
Keywords: Adult, ADHD, eHealth, Super Brains

Conflict of interest:

No

Article ID: S045
Subjects: Abstract, Immune Alterations in Early Psychosis: New Goals for Diagnosis and Treatment

Kynurenine pathway, inflammation and schizophrenia

Szulc A.
Medical University of Warsaw , Department of Psychiatry, Pruszków , Poland
Keywords: kynurenine pathway, schizophrenia

Conflict of interest:

No

Article ID: S048

Unravelling immune alterations associated with the deficit schizophrenia subtype

Samochowiec J. 1 *
Misiak B. 1 2
Kucharska-Mazur J. 1
Wroński M. 1
1 Pomeranian Medical University, Department of Psychiatry , Szczecin , Poland
2 Medical University Wrocław, Genetics , Wroclaw , Poland
* Corresponding author.
Keywords: deficite schizophrenia, Neural developement, neuroinflammation, biological marker

Conflict of interest:

No

Article ID: S051
Subjects: Abstract, Psychiatric Comorbidities in Bipolar Disorders: Double Penalty and Trajectories

The role of substance abuse and polygenic risk in the early course of bipolar disorder

Lagerberg T.V.
Oslo University Hospital , Coe Norment Centre For Mental Disorders Research, Oslo , Norway
Keywords: Bipolar disorder, substance abuse, Polygenic risk, Clinical outcome

Conflict of interest:

No

Article ID: S052

Psychopathological outcomes of clinically relevant adolescent borderline personality disorder symptoms

Marwaha S.
University of Birmingham , Institute For Mental Health, School of Psychology, Birmingham , United Kingdom
Keywords: Hypomania, Borderline Personality Disorder, adolescents, Outcomes

Conflict of interest:

No

Article ID: S053
Subjects: Abstract, Psychosocial Imaging: Disentangling the Interplay between Environmental Variables and Psychotic Disorders

The causal role of environmental factors in psychotic disorders with a focus on urban living

Kirkbride J.
University College London , Division of Psychiatry, London , United Kingdom
Keywords: epidemiology, psychotic Disorders, causal inference, social determinants

Conflict of interest:

No

Article ID: S054

The complexity of vulnerability to psychosis

Fiorillo A.
University of Campania "L. Vanvitelli" , Department of Psychiatry, Naples , Italy
Keywords: psychosis, risk factors, complexity

Conflict of interest:

No

Article ID: S057
Subjects: Abstract, Bipolar Disorders on Top of Mood Stabilisers: Can We Improve the General Health?

Focus on metabolic features associated with bipolar disorders

Jaworowski S. 1
Pacchiarotti I. 2 *
1 Shaare Zedek Medical Centre, Consultation Liaison Psychiatry , Jerusalem , Israel
2 Bipolar and Depressive Disorders Unit, Hospital Clínic de Barcelona, Psychiatry , Barcelona , Spain
* Corresponding author.
Keywords: Bipolar Disorder, Health, metabolic syndrome, treatment

Article ID: S058

Innocence faded: childhood adversity and the hippocampus in healthy and bipolar individuals

Janiri D.
Sapienza University , Psychiatry, Rome , Italy
Keywords: Bipolar Disorders, childhood trauma, Hippocampus

Conflict of interest:

No

Article ID: S059

Sleep in bipolar disorders: a prodrome, a symptom, a treatment?

Murru A.
Bipolar and Depressive Disorders Unit, Hospital Clínic de Barcelona, Psychiatry , Barcelona , Spain
Keywords: Bipolar disorder, sleep, Dépression, prodrome

Conflict of interest:

No

Article ID: S060

Psychosocial interventions for patients with bipolar disorder and their relatives

Fiorillo A. 1
Luciano M. 2 *
1 University of Campania "L. Vanvitelli" , Department of Psychiatry, Naples , Italy
2 University of Campania "Luigi Vanvitelli", Department of Psychiatry , Naples , Italy
* Corresponding author.
Keywords: Psychosocial intervention, family burden, family psychoeducational intervention

Conflict of interest:

No

Article ID: S061
Subjects: Abstract, Negative Symptoms in Subjects with Schizophrenia and at High Risk for Psychosis: The State of the Art of Assessment and Treatment

Main advantages and limitations of validated assessment instruments for negative symptoms

Dollfus S. 1 2
1 UNICAEN, Normandie Université , Ists, Ea7466, Caen , France
2 CHU de Caen, Department of Psychiatry , Caen , France
Keywords: schizophrenia, negative symptoms, assessment, self-assessment

Article ID: S064

Evidence-based treatment of negative symptoms?

Kaiser S.
University of Geneva Hospitals , Adult Psychiatry Division, Department of Psychiatry, Geneva , Switzerland
Keywords: schizophrenia, negative symptoms, treatment

Article ID: S067
Subjects: Abstract, Intercultural Opening of Mental Health Care Services across Europe

Do asylum seekers and refugees need specialised services? pitfalls and challenges of mental health care for asylum seekers and refugees. status quo in europe

Küey L.
Istanbul Bilgi University , Psychology, Istanbul , Turkey
Keywords: Mainstream services, mental health care, Refugees and asylum seekers, Special services

Conflict of interest:

No

Article ID: S072
Subjects: Abstract, My Dealer Is a Doctor: On Latrogenic Addictions

Should we stimulate prescribing stimulants?

Ramos-Quiroga J.A.
Hospital Universitari Vall d'Hebron. Universitat Autònoma de Barcelona, Department of Psychiatry , Barcelona , Spain

Article ID: S073
Subjects: Abstract, Unconventional Treatments with Unique Mechanistic Targets for Major Depressive Disorder

Unconventional treatments with unique mechanistic targets for major depressive disorder: ketamine

Vazquez G.
McLean Hospital, Harvard Medical School , International Consortium For Bipolar & Psychotic Disorders Research, Boston , United States of America
Keywords: Treatment Resistant Depresion, NMDA Receptor, ketamine, Suicide

Conflict of interest:

No

Article ID: S075

Unconventional treatments with unique mechanistic targets for major depressive disorder: cannabinoids

Bahji A.
Providence Care Hospital, Queen's University, Psychiatry , Kingston , Canada
Keywords: Cannabinoids, Depressive Disorder, Major

Conflict of interest:

No

Article ID: S082
Subjects: Abstract, Major Depression as an Episodic Illness: Benefits of Re-Framing the Disease Course

Heterogeneity of major depressive disorder versus postpartum depression

Kasper S.
Medical University of Vienna , Center of Brain Research, Vienna , Austria
Keywords: major depressive disorder, postpartum depression, heterogeneity

Article ID: S083

Why do we consider major depressive disorder to be a chronic illness?

Vieta E.
University of Barcelona , Department of Psychiatry and Psychology, Barcelona , Spain
Keywords: major depressive disorder, diagnosis, treatment, Antidepressants

Article ID: S084

Sage-217 clinical data in patients with a depressive episode in major depressive disorder

Lasser R.
Sage Therapeutics, Inc., Department of Medical Science , Cambridge , United States of America
Keywords: major depressive disorder, SAGE-217, GABA

Article ID: S085

Brexanolone and sage-217 clinical data in patients with postpartum depression

Deligiannidis K.
Zucker Hillside Hospital, Northwell Health, Psychiatry , Glen Oaks , United States of America
Keywords: SAGE-217, brexanolone, postpartum depression, GABA

Article ID: S087
Subjects: Abstract, Suicide Behaviour in Different Mental Disorders: Epidemiological Findings and Perspectives on Suicide Prevention

Prevalence and incidence of suicidal ideation and behaviour in delusional disorder, schizophrenia and other related disorders

González-Rodríguez A.
Parc Taulí University Hospital. Autonomous University of Barcelona (UAB). I3PT , Mental Health, Sabadell , Spain
Keywords: Delusional disorder, Suicide, Suicidal ideation, psychosis

Article ID: S088

Pain and interoception: perspectives on suicide prevention

Courtet P.
University of Montpellier. , Department of Emergency Psychiatry & Acute Care, Montpellier , France
Keywords: Suicidal behaviour, interoception, Psychological pain, Inflammation

Conflict of interest:

No

Article ID: S089

Effectiveness of suicide prevention programs at emergencies and community mental health services

Vidal D. Palao 1 2
1 Parc Tauli-University Hospital, Mental Health , Sabadell (Barcelona), Spain
2 Parc Tauli-University Hospital, Mental Health. Cibersam , Sabadell (Barcelona), Spain
Keywords: Catalonia Suicide Risk Code, Suicide prevention, Effectiveness

Conflict of interest:

No

Article ID: S099
Subjects: Abstract, Mental Health and the Transition from Child to Adult Mental Health Services in Europe

The black hole of care process before the age of transition

Reneses B.
San Carlos University Hospital, Institute of Psychiatry and Mental Health , Madrid , Spain
Keywords: Transition, health services research, adolescent mental care pathways, Youth Mental Health

Conflict of interest:

No

Article ID: S103
Subjects: Abstract, Perinatal Depression: Psychopathological Characteristics and Treatment Strategies

Is pharmacotherapy safe in the perinatal period?

Wieck A.
University of Manchester & Wythenshawe Hospital , Greater Manchester Mental Health Foundation Trust, Manchester , United Kingdom

Conflict of interest:

No

Article ID: S105

Development and efficacy of a new psychoeducational intervention for peripartum depression

Fiorillo A.
Luciano M. *
University of Campania "L. Vanvitelli" , Department of Psychiatry, Naples , Italy
* Corresponding author.
Keywords: Perinatal Depression, Psychosocial intervention, coping strategies

Conflict of interest:

No

Article ID: S118
Subjects: Abstract, The Ageing Schizophrenia Population: A New Growing Population with New Challenges

Clinical evolution of schizophrenia in old age

Von Gunten A.
Centre hospitalier universitaire vaudois (CHUV), Service Universitaire De Psychiatrie De L’âge Avancé (supaa), Prilly , Switzerland
Keywords: old age, schizophrenia, Clinical evolution

Conflict of interest:

No

Article ID: S121

Long-term antipsychotic treatment in patients with schizophrenia: the gap between research and practice

Stuhec M.
University of Ljubljana , Faculty of Pharmacy & Ormoz Psychiatric Hospital, Clinical Pharmacy, Ormoz, Slovenia
Keywords: Clinical trials, psychopharmacology, antipsychotics, meta-analysis

Conflict of interest:

No

Article ID: S123
Subjects: Abstract, Current Challenges in the Diagnosis and Treatment of Behavioural Addictions

Compulsive buying: is there enough evidences to recognize it as a disorder?

Claes L.
University of Leuven , Psychology And Educational Sciences, Leuven , Belgium
Keywords: compulsive buying, ETIOLOGY, disorder, prevalence

Conflict of interest:

No

Article ID: S128
Subjects: Abstract, New Ideas and Technologies in the Treatment of Anxiety and Psychotic Disorders

Cognitive behavioural therapy augmented with virtual reality exposure for treatment of social anxiety: preliminary results from a qualitative pilot

Thorup B. *
Bang P.
Mental Health Center Copenhagen, Copenhagen Research Center For Mental Health – Core , Hellerup , Denmark
* Corresponding author.
Keywords: Social Anxiety Disorder, qualitative, Virtual Reality Exposure, Pragmatic

Conflict of interest:

No

Article ID: S130
Subjects: Abstract; Treating Anxiety, Depression and Bipolar Disorders: Aiming at the Amygdala

Anxiety, mindfulness and the amygdala

Piguet C.
UNIGE, Psychiatry Department , Geneva , Switzerland

Conflict of interest:

No

Article ID: S131

Role of insula to amygdala projection neurons in anxiety- and valence-related behaviors

Beyeler A.
INSERM 1215, Neurocentre Magendie, Univ. De Bordeaux, Bordeaux , France
Keywords: Circuit, optogenetics, SEROTONIN, calcium imaging

Conflict of interest:

No

Article ID: S133

Fmri based neurofeedback of the amygdala in bipolar disorder

Favre P. 1 2
1 CEA, Neurospin Neuroimaging Platform, Gif Sur Yvette , France
2 INSERM U955, Team 15 "translational Psychiatry", Créteil, France
Keywords: Bipolar disorder, neurofeedback, fMRI, amygdala

Conflict of interest:

No

Article ID: S139
Subjects: Abstract, Can Quality Assurance of Psychiatric Services Reduce Stigma and Discrimination?

Quality assurance: systemic processes and information to obtain consent

Akiyama T.
NTT Medical Center, Neuropsychiatry , Tokyo , Japan
Keywords: self-stigma, quality assurance, systemic process, information for consent

Conflict of interest:

No

Article ID: S141

The contribution of quality medicine to overcoming stigma and discrimination of people with mental illness

Gaebel W.
Heinrich-Heine-Universität Düsseldorf, Department of Psychiatry and Psychotherapy, Lvr Klinikum , Düsseldorf , Germany
Keywords: quality, Stigma

Conflict of interest:

No

Article ID: S142
Subjects: Abstract, Are Psychodelics Useful in the Treatment of Mental Disorders?

Psychedelics revisited: therapeutic potential of pharmacological outcasts

Mohr P. 1 2
1 3rd Faculty of Medicine, Charles University , Psychiatric Clinic, Praha , Czech Republic
2 National Institute of Mental Health , Klecany, Clinical Dept., Klecany , Czech Republic
Keywords: Psychedelics, pharmacology, pharmacotherapy

Conflict of interest:

No

Article ID: S143

Functional changes induced by psychodelic drugs

Preller K.
University of Zürich , Department of Psychiatry, Pychotherapy And Psychosomatics, Zürich , Switzerland
Keywords: fMRI, neuroimaging, Psychedelics, SEROTONIN

Conflict of interest:

No

Article ID: S146
Subjects: Abstract, Current Picture and Future Directions of Child and Adolescent Psychiatry Training in Europe

Cap in gap: a look at the quality of cap training for adult psychiatry trainees

Seker A.
Erciyes University Hospital , Child and Adolescent Psychiatry, Kayseri , Turkey
Keywords: rotation, Child and adolescent psychiatry, CAMHS, cap

Conflict of interest:

No

Article ID: S148

Current challenges and opportunities in cap training throughout europe: a trainee perspective

Mazaliauskienė R. 1
Gnanavel S. 2 *
1 Republic Kaunas Hospital’s Division, Psychiatric Clinic Of Luhs , Kaunas , Lithuania
2 Health Education North East England, Child and Adolescent Psychiatry , Newcastle upon Tyne , United Kingdom
* Corresponding author.
Keywords: trainee perspective, CAP training, Europe

Conflict of interest:

No

Article ID: S151
Subjects: Abstract, Improving Lifestyle Behaviours in People with Severe Mental Disorders

Reducing mortality gap in schizophrenia: healthy lifestyles and pharmacological treatment

Pompili M.
Sapienza University of Rome , Neurosciences, Mental Health and Sensory Organs, Rome , Italy
Keywords: Suicide, Lifestyles, mortality, schizophrenia

Article ID: S152

Can psychosocial treatment reduce antipsychotic-induced metabolic disturbances in patients with first episode psychosis?

Rojnic-Kuzman M. 1 *
Kuharic D. Bosnjak 2
Kekin I. 1
Makaric P. 2
Gajsak L. Rossini 3
Madzarac Z. 4
Makar A. Koricancic 4
Vogrinc Z. 4
1 Zagreb School of Medicine end ZagUniversity Hospital Centre , Department of Psychiatry, Zagreb , Croatia
2 University Psychiatric Hospital Vrapce , Department For Diagnostics And Intesive Care, Zagreb , Croatia
3 Neuropsyhiatric Hospital, Department of Biological Psychiatry, Popovaca , Croatia
4 University Hospital Centre Zagreb , Psychiatry, Zagreb , Croatia
* Corresponding author.
Keywords: DAY HOSPITAL, metabolic syndrome, First Episode Psychosis, Psychosocial intervention

Article ID: S153

The role of psycho-educational interventions in improving physical health of people with severe mental illness

Fiorillo A.
Sampogna G. *
University of Campania "L. Vanvitelli" , Department of Psychiatry, Naples , Italy
* Corresponding author.
Keywords: comorbidity, mortality gap, lifestyle

Conflict of interest:

No

Article ID: S154
Subjects: Abstract, Internet interventions with CBT

Scientific basis for ICBT

Berger T.
Klinische Psychologie und Psychotherapie, Universität Bern , Bern , Switzerland
Keywords: Internet, CBT, iCBT

Conflict of interest:

No

Article ID: S160
Subjects: Abstract, Non-Invasive brain stimulation for late life depression: challenges and perspectives

NIBS for TRD in the elderly: dealing with comorbidities

Calvet B.
Esquirol hospital center, Old Age Psychiatry , Limoges , France
Keywords: comorbidities, drug-resistant depression, non-invasive brain stimulation, stimulation parameters

Conflict of interest:

No

Article ID: S166
Subjects: Abstract, Can Innovative Technologies Optimise Mental Health Care? Some Shining Examples

Virtual reality in primary prevention: bullying in the classroom

Barreda M. 1 *
Serra-Blasco M. 2
Cano-Català M. 2
Pereda-Baños A. 3
Goldberg X. 2
Álvarez N. Cardoner 4
1 Vrije University , Communication Science, HV Amsterdam , Netherlands
2 Parc Taulí Hospital Universitari, Mental Health , Sabadell , Spain
3 Eurecat, Data Science , Barcelona , Spain
4 University Hospital Parc Taulí , Mental Health, Sabadell , Spain
* Corresponding author.
Keywords: school bullying, virtual reality, attitudes, Empathy

Conflict of interest:

No

Article ID: S169

When digital technologies met neuroimaging: implications for clinical psychiatry

Soriano-Mas C.
Bellvitge University Hospital-IDIBELL , Psychiatry, Barcelona , Spain
Keywords: Neuroimaging, Digital Technologies, Machine Learning, Biological Psychiatry

Conflict of interest:

No

Article ID: S178
Subjects: Abstract, News and mechanism of action (MOA) of ECT in depression

The truth about electroconvulsive therapy: a new era of knowledge. clinical predictors of therapeutic response to electroconvulsive therapy

Iglesias M. 1 *
Cardoner N. 2
1 Hospital German Trias i Pujol, Psychiatry , Badalona , Spain
2 Consorci Corporació Sanitària Parc Taulí, Psychiatry , Sabadell , Spain
* Corresponding author.
Keywords: Electroconvulsive Therapy, ECT, Dépression, CLINICAL PREDICTORS

Conflict of interest:

No

Article ID: S180

Mechanism of action of electroconvulsive therapy: a multimodal neuroimaging model

Cano-Catala M.
University Hospital Parc Taulí , Mental Health, Sabadell , Spain
Keywords: Electroconvulsive Therapy, Treatment-Resistant Depression, Magnetic Resonance Imaging

Conflict of interest:

No

Article ID: S181

Gemric: the global ect-mri research collaboration

Oltedal L. 1 2
1 University of Bergen , Clinical Medicine, Bergen , Norway
2 Haukeland University Hospital , Mohn Medical Imaging And Visualization Centre, Bergen , Norway
Keywords: Dépression, Electroconvulsive Therapy, Magnetic Resonance Imaging

Conflict of interest:

No

Article ID: S182
Subjects: Abstract, Adult Offenders and Child Victims of Sexual Abuse: An Update for Psychiatrists

General introduction to sex offending emphasising prevalence, diagnostics and risk assessment and risk management

Goethals K. 1 2
1 Directeur Universitair Forensisch Centrum (UFC), forensisch psychiater, Universitair Ziekenhuis Antwerpen, Universitaire Ziekenhuisdienst Psychiatrie , Edegem , Belgium
2 University of Antwerp , Capri, Wilrijk , Belgium
Keywords: sex offending, prevalence, Diagnostics, Risk assessment

Conflict of interest:

No

Article ID: S184

Consequences of child abuse and neglect in adulthood

Plener P.
Medical University of Vienna , Department of Child and Adolescent Psychiatry, Vienna , Austria
Keywords: child neglect, adverse childhood experience, Child abuse, trauma

Conflict of interest:

No

Article ID: S187
Subjects: Abstract, Identifying and Targeting Suicide Risk Factors in Youth

Protective and risk-conferring factors for suicidality in youth in europe

Sarchiapone M. 1 2
1 National Institute for Health , Migration and Poverty, Nihmp, Rome , Italy
2 Università degli Studi del Molise, Department of Medicine end Health Sciences , Molise , Italy
Keywords: Suicide, adolescents

Article ID: S188

Perspectives of modern society in youth suicide prevention

Pompili M.
Sapienza University of Rome , Neurosciences, Mental Health and Sensory Organs, Rome , Italy
Keywords: youths, Suicide, Prevention

Conflict of interest:

No

Article ID: S189

Treating and preventing suicidality in youth: assessing the evidence for efficacy

Wasserman D.
Karolinska Institutet, National Centre For Suicide Research and Prevention of Mental Lll-health , Stockholm , Sweden
Keywords: Youth, Prevention, treatment, Evidence

Conflict of interest:

No

Article ID: S193
Subjects: Abstract, More Treatment but Not Less Depression: The Paradox of over- and under-Diagnosis in Depression

More treatment but not less depression: reasons and remedies

Ormel J.
University Medical Center Groningen , Department of Psychiatry, Groningen , Netherlands

Conflict of interest:

No

